# Delta/Jagged-mediated Notch signaling induces the differentiation of *agr2*-positive epidermal mucous cells in zebrafish embryos

**DOI:** 10.1371/journal.pgen.1009969

**Published:** 2021-12-28

**Authors:** Yu-Fen Lu, Da-Wei Liu, I-Chen Li, Jamie Lin, Chien-Ming Wang, Kuo-Chang Chu, Hsiao-Hui Kuo, Che-Yi Lin, Ling-Huei Yih, Yun-Jin Jiang, Sheng-Ping L. Hwang

**Affiliations:** 1 Institute of Cellular and Organismic Biology, Academia Sinica, Taipei, Taiwan; 2 Institute of Molecular and Genomic Medicine, National Health Research Institutes, Zhunan, Taiwan; 3 Biotechnology Center, National Chung Hsing University, Taichung, Taiwan; 4 Department of Bioscience and Biotechnology, National Taiwan Ocean University, Keelung, Taiwan; University of Pennsylvania School of Medicine, UNITED STATES

## Abstract

Teleosts live in aquatic habitats, where they encounter ionic and acid-base fluctuations as well as infectious pathogens. To protect from these external challenges, the teleost epidermis is composed of living cells, including keratinocytes and ionocytes that maintain body fluid ionic homeostasis, and mucous cells that secret mucus. While ionocyte progenitors are known to be specified by Delta-Notch-mediated lateral inhibition during late gastrulation and early segmentation, it remains unclear how epidermal mucous cells (EMCs) are differentiated and maintained. Here, we show that Delta/Jagged-mediated activation of Notch signaling induces the differentiation of *agr2*-positive (*agr2*^*+*^) EMCs in zebrafish embryos during segmentation. We demonstrated that *agr2*^+^ EMCs contain cytoplasmic secretory granules and express *muc5*.*1* and *muc5*.*2*. Reductions in *agr2*^+^ EMC number were observed in *mib* mutants and *notch3* MOs-injected *notch1a* mutants, while increases in *agr2*^+^ cell number were detected in *notch1a-* and *X-Su(H)/ANK*-overexpressing embryos. Treatment with γ-secretase inhibitors further revealed that Notch signaling is required during bud to 15 hpf for the differentiation of *agr2*^+^ EMCs. Increased *agr2*^+^ EMC numbers were also observed in *jag1a*-, *jag1b*-, *jag2a*- and *dlc*-overexpressing, but not *jag2b*-overexpressing embryos. Meanwhile, reductions in *agr2*^+^ EMC numbers were detected in *jag1a* morphants, *jag1b* mutants, *jag2a* mutants and *dlc* morphants, but not *jag2b* mutants. Reduced numbers of *pvalb8*-positive epidermal cells were also observed in *mib* or *jag2a* mutants and *jag1a* or *jag1b* morphants, while increased *pvalb8*-positive epidermal cell numbers were detected in *notch1a-*overexpressing, but not *dlc*-overexpressing embryos. BrdU labeling further revealed that the *agr2*^+^ EMC population is maintained by proliferation. Cell lineage experiments showed that *agr2*^*+*^ EMCs are derived from the same ectodermal precursors as keratinocytes or ionocytes. Together, our results indicate that specification of *agr2*^+^ EMCs in zebrafish embryos is induced by DeltaC/Jagged-dependent activation of Notch1a/3 signaling, and the cell population is maintained by proliferation.

## Author summary

As aquatic organisms, fish must tolerate environmental challenges that include acid-base fluctuations and water-borne pathogens. The skin provides a first line of defense against these challenges, and specific cell types in the tissue are responsible for different protective functions. For example, keratinocytes provide body coverage, ionocytes are responsible for maintaining body fluid ionic homeostasis, and epidermal mucous cells generate a protective layer of mucus that covers the entire fish surface. In this study, we uncovered the developmental process in zebrafish that underlies the generation of epidermal mucous cells. First, we characterized epidermal mucous cells according to their expression of a particular gene, *agr2*. Then, we found that these cells differentiate soon after ionocytes and keratinocytes, and the molecular pathways that guide differentiation of all three cell types involve similar signals. While ionocytes and keratinocytes are known to be specified by Delta-Notch-mediated lateral inhibition, we found that epidermal mucous cells are specified by activation of Notch by Delta and Jagged ligands. Thus, our results suggest that the specification of these major cell types in the epidermis occurs via a streamlined Notch-dependent process. This utilization of temporally distinct signaling events can therefore generate diverse cell types in the fish epidermis.

## Introduction

Mammalian skin is composed of four stratified epithelial layers, including the stratum basale, stratum spinosum, stratum granulosum and the outermost stratum corneum [1]. Notably, the stratum corneum consists of layers of dead corneocytes that serve as an inert hydrophobic boundary between the animal and the environment. Together the stratified epithelia of skin epidermis form a complex barrier that protects terrestrial vertebrates from dehydration, pathogen infection and mechanical stress. Several transcription factors and signaling pathways (p63, KLF4, IRF6, Grhl3, MAPK cascade and Notch signaling) have been implicated in epidermal stratification, renewal and differentiation [2–8].

In contrast to mammalian species, which are mostly terrestrial, teleosts live in aquatic habitats, where they encounter ionic, osmotic and acid-base fluctuations as well as infectious pathogens. To cope with these environmental challenges, teleost skin is composed of living cells covered with a layer of mucus [9]. In zebrafish, the embryonic epidermis consists of a surface enveloping layer, which develops into periderm, and an inner epidermal basal layer containing both undifferentiated and differentiated cells (ionocytes and mucous cells) [10]. In the adult zebrafish, the epidermis consists of three layers: a surface layer of keratinocytes, an intermediate layer composed of ionocytes, mucous cells, club cells and undifferentiated cells, and a basal layer of undifferentiated cells.

In the skin of embryos and the gills of adult fish, specialized ionocytes serve to maintain body fluid ionic homeostasis [11,12]. Both ionocytes and keratinocytes are derived from common precursors, which are located in the ventral non-neural ectoderm and express a dominant-negative form of p63 (△Np63) at the late gastrula stage of zebrafish embryos [13]. Whether cells from p63^+^ ventral ectoderm become ionocytes or keratinocytes is determined by Delta-Notch lateral inhibition [14,15]. Epidermal cells that express high levels of Dlc ligand develop into ionocyte progenitors. The Dlc ligand interacts with Notch1a/3 receptors on adjacent epidermal cells to prevent *dlc* expression in those cells, causing them to become keratinocytes. Notably, the ionocyte progenitor population was recently shown to be maintained by Klf4 via its regulation of p63^+^ epidermal stem cell proliferation and DeltaC-Notch lateral inhibition [16]. The development of different types of ionocytes, including Na^+^, K^+^-ATPase-rich cells (NaRCs) and H^+^-ATPase-rich cells (HRCs), is further mediated by the *foxi3a*/*foxi3b* regulatory loop [14].

A third type of epidermal cells is mucous cells. The major components of mucus are mucous cell-secreted Mucin glycoproteins, which form a network of polymers joined by disulfide bridges [17]. Innate immune-related proteins (antimicrobial peptide and apolipoprotein A-I) are also found in fish epidermal mucus [18–20]. Thus, mucus is considered to be the first line of defense against pathogen invasion; however, it remains unclear how epidermal mucous cells (EMCs) develop in fish. A recent study showed that *pvalb8* (a putative EMC marker), *atp1b1b* (a NaRC marker) and *atp6v1aa* (a HRC marker, previously named *atp6v1al*) display complementary punctate expression patterns in zebrafish embryos at 24 hours post-fertilization (hpf) [21]. Furthermore, knockdown of *foxi3a* caused loss of *atp1b1b*^*+*^ ionocytes and expansion of *pvalb8*-positive cells, suggesting that *pvalb8*-positive cells and ionocytes are derived from common non-keratinocyte precursors [21]. Previously, we found that the gene, anterior gradient 2 (*agr2*), is expressed in most zebrafish organs that contain mucus-secreting cells, including the intestine and epidermis of embryos [22]. Importantly, knockdown of *agr2* was shown to disrupt terminal differentiation of intestinal goblet cells in zebrafish embryos [23], and maturation of intestinal goblet cells is known to be regulated by Foxa2 and Hif1ab-induced *agr2* expression [24]. We were therefore interested to determine whether *agr2*^+^ EMCs are derived from non-keratinocyte precursors, like *pvalb8*-positive cells.

In this study, we first demonstrated that *agr2*^+^ EMCs contain cytoplasmic secretory granules and express *muc5*.*1* and *muc5*.*2*. Then, we found that the numbers of *agr2*^+^ EMCs were reduced in *mib* mutants and *notch3* MOs-injected *notch1a* mutants. On the other hand, the *agr2*^+^ EMC numbers were increased in *notch1a*- and *X-Su(H)/ANK*-overexpressing embryos. Treatment with γ-secretase inhibitors further revealed that Notch signaling is required during bud to 15 hpf for the differentiation of *agr2*^+^ EMCs. Next, we determined that *agr2*^+^ EMC numbers were increased in *jag1a*-, *jag1b*-, *jag2a*- and *dlc*-overexpressing, but not *jag2b*-overexpressing embryos, while *agr2*^+^ EMC numbers were diminished in *jag1a* morphants, *jag1b* mutants, *jag2a* mutants and *dlc* morphants, but not *jag2b* mutants. Reductions in *pvalb8*-positive epidermal cell numbers were also observed in *mib* mutants, *jag1a* morphants, *jag1b* morphants and *jag2a* mutants, while increased *pvalb8*-positive epidermal cell numbers were detected in *notch1a-*overexpressing, but not *dlc*-overexpressing embryos. BrdU labeling was then used to show that the *agr2*^+^ EMC population is maintained by proliferation. Cell lineage experiments further demonstrated that *agr2*^*+*^ EMCs and keratinocytes or ionocytes are derived from common ectodermal precursors.

## Results

### Development of *agr2*-expressing EMCs

**Fig 1:**
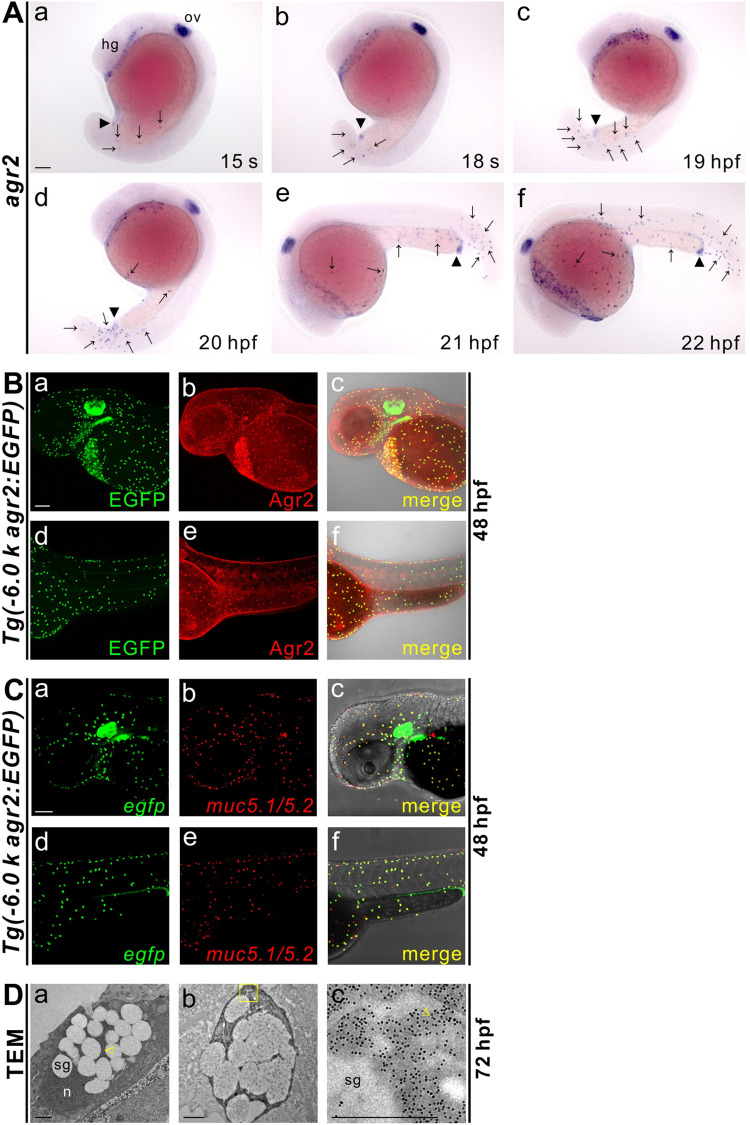
Characterization of *agr2*^*+*^ epidermal mucous cells. **(A).** Developmental expression pattern of *agr2* in the epidermis from 15 s to 22 hpf. hg, hatching gland; ov, otic vesicle. Arrows, EMCs. Arrowhead, anus. Scale bar, 100 μm. **(B).** Colocalization of Agr2 (red) and EGFP (green) was detected in the trunk and yolk regions of *Tg(-6*.*0 k agr2*:*EGFP)* embryos at 48 hpf. Scale bar, 100 μm. **(C).** *egfp* (green) is coexpressed with *muc5*.*1*/*muc5*.*2* (red) in *Tg(-6*.*0 k agr2*:*EGFP)* embryos at 48 hpf. Scale bar, 100 μm. **(D).** Ultrastructure of EMCs and GFP immuno labeling in *Tg(-6*.*0 k agr2*:*EGFP)* embryos at 72 hpf. n, nucleus; sg, secretory granules. Yellow arrowhead, endoplasmic reticulum. Yellow square box indicates enlarged area. Scale bars, 1 μm. Scale bar for enlarged image (c), 0.5 μm.

Initially, we conducted *in situ* hybridization to find the earliest stage at which *agr2*^*+*^ EMCs emerge. At 15 s, *agr2* began to be expressed in the epidermis of the developing yolk extension, in addition to its expression in the otic vesicles, hatching gland and future anus (Fig 1Aa). Increased signal for *agr2*^*+*^ EMCs was then detected in both tail and yolk extension during 18 s and 19 hpf stages (Fig 1Ab and 1Ac). *Agr2*^*+*^ EMCs were detected in the yolk sac from 20 hpf and in the trunk, tail and different yolk regions at 22 hpf (Fig 1Ad-1Af).

Previously, we investigated the mechanisms controlling *agr2* expression in the pharynx and intestine of zebrafish; in doing so, we generated a *Tg(-6*.*0 k agr2*:*EGFP)* transgenic line, which recapitulated endogenous *agr2* expression [24]. In order to confirm whether EGFP in the reporter line colocalizes with Agr2 in EMCs, we performed double immunofluorescence staining using anti-salmon Agr2 and anti-GFP antibodies. Colocalization of Agr2 and EGFP was detected in the EMCs within the yolks and trunks of *Tg(-6*.*0 k agr2*:*EGFP)* transgenic embryos at 48 hpf (Fig 1B).

In previous studies, *pvalb8-*positive cells were identified as putative EMCs in zebrafish embryos [21,25]. We therefore wondered whether *agr2* and *pvalb8* are co-expressed in the same epidermal cells. To answer this question, we conducted double fluorescence *in situ* hybridization on *Tg(-6*.*0 k agr2*:*EGFP)* transgenic embryos. Distinct epidermal cells expressing *egfp/agr2* or *pvalb8* were identified in the trunk and yolk sac at 24 hpf, indicating that *agr2*^*+*^ EMCs and *pvalb8*-positive cells may represent two different populations of EMCs (S1 Fig).

Since intestinal goblet cells mostly secrete Muc2 glycoprotein to create a mucus layer [26], we investigated which mucin genes are expressed in the EMCs. We found that *muc5*.*1/5*.*2* were co-expressed with *egfp* in the EMCs within the trunks and yolks of *Tg(-6*.*0 k agr2*:*EGFP)* embryos at 48 hpf (Fig 1C).

To examine the ultrastructure of EMCs, we conducted transmission electron microscopy analyses and immunogold labeling on 72 hpf *Tg(-6*.*0 k agr2*:*EGFP)* embryos (Fig 1D). The cytoplasm of each EMC was filled with secretory granules surrounded by endoplasmic reticulum, and the nucleus was located at one side of the cell (Fig 1Da). GFP immunogold electron microscopy staining showed that gold-labeled GFP was distributed in the rough endoplasmic reticulum and lumen, cytoplasm and secretory granules (Fig 1Db and 1Dc). Together, these results indicated that *agr2*^*+*^ EMCs develop during mid-segmentation stages and secrete Muc5.1/5.2 glycoprotein to form a mucus layer. Moreover, *agr2*^*+*^ EMCs and *pvalb8*-positive cells may represent two different populations of EMCs.

### Proper Notch signaling is required for the differentiation of *agr2*^*+*^ EMCs

**Fig 2:**
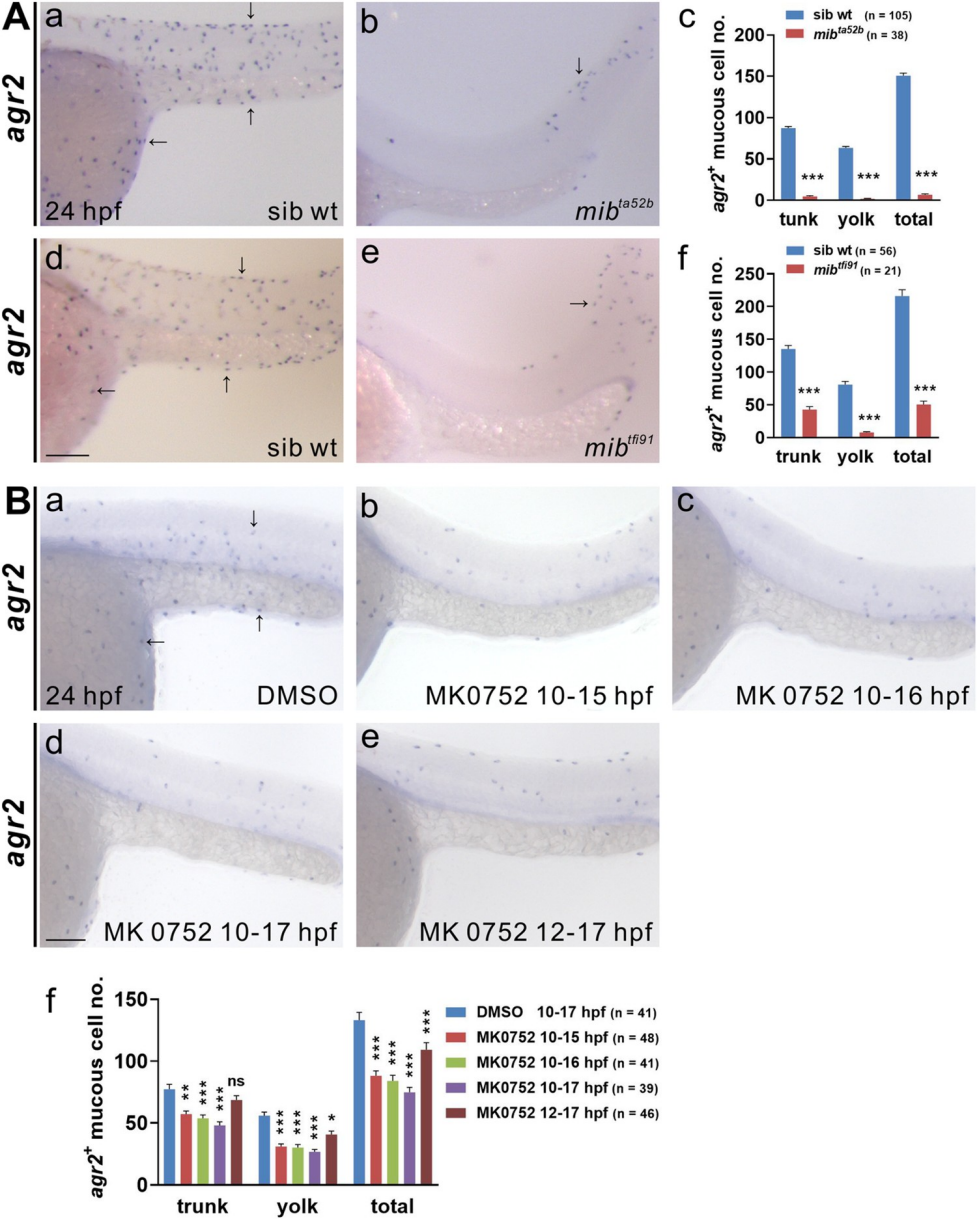
Notch signaling is required for the differentiation of *agr2*^*+*^ epidermal mucous cells. **(A).**Significant reductions in *agr2*^*+*^ EMC numbers were detected in two *mib* homozygous mutants compared to sibling wild-type embryos at 24 hpf. **(B).** Larger reductions in *agr2*^*+*^ EMC numbers were detected in embryos treated with γ-secretase inhibitor (MK0752) from 10–15 hpf, 10–16 hpf and 10–17 hpf than in those treated from 12–17 hpf. Arrows, EMCs. Scale bars, 100 μm. Mean ± SEM. Student’s *t*-test. *p<0.05; **p<0.01; ***p<0.001; ns, not significant. Underlying data are available in S1 Data.

Since Delta-Notch-mediated lateral inhibition determines the cell fates of ionocytes and keratinocytes [14,15], we wondered whether Notch signaling is also involved in the development of *agr2*^*+*^ EMCs. We conducted *in situ* hybridization using *agr2* RNA probes and observed a significant reduction in *agr2*^*+*^ EMC numbers in the yolks and trunks of *mib*^*ta52b*^ and *mib*^*tfi91*^ mutants compared to respective sibling wild-type embryos at 24 hpf (Fig 2Aa-2Af). Of note, the reduction of *agr2*^*+*^ EMC number was higher for *mib*^*ta52b*^ mutants (95.6%) than that for *mib*^*tfi91*^ mutants (76.6%), which is consistent with the fact that *mib*^*ta52b*^ is an antimorphic allele [27]. Additionally, we observed a substantial decrease in *pvalb8*-positive cell number in the yolks and trunks of *mib*^*ta52b*^ and *mib*^*tfi91*^ mutants compared to respective sibling wild-type embryos at 24 hpf, although reduction levels were less than those of *agr2*^*+*^ EMC number (S2A Fig).

We next investigated the timing at which *agr2*^*+*^ EMCs require Notch signaling. We incubated zebrafish embryos with a γ-secretase inhibitor (MK0752) at different developmental stages to evaluate the effects on *agr2*^*+*^ EMCs. Initially, we treated embryos with 100 μM MK0752 for the intervals of bud (10 hpf)-17 hpf, 10–20 hpf or 10–24 hpf. We observed substantial reductions in *agr2*^*+*^ EMC numbers in the trunks and yolks compared to embryos treated with DMSO from 10–24 hpf (S2B Fig). In order to find the required minimal treatment time, we treated embryos with 50 μM MK0752 from 10–13 and 10–14 hpf or 11–14 and 11–15 hpf. We found that 3 h of MK0752 treatment did not affect *agr2*^*+*^ EMC number in the trunks and yolks, whereas 4 h of MK0752 treatment significantly reduced *agr2*^*+*^ EMC numbers in these regions compared to DMSO-treated embryos (S2C Fig). We then treated embryos with 75 μM MK0752 from 10–15, 10–16 and 10–17 hpf and observed incremental reductions of *agr2*^*+*^ EMC numbers in the trunks and yolks compared to embryos treated with DMSO from 10–17 hpf (Fig 2B). Substantial decreases were seen in total *agr2*^*+*^ EMC number and EMC number in the yolk (but not in the trunk) in 12–17 hpf-treated embryos; however, these reductions were less than those detected in MK0752-treated embryos from 10–15 hpf. Together, these results indicate that Notch signaling is essential for the development of *agr2*^*+*^ EMCs and *pvalb8*-positive cells. Furthermore, Notch signaling is required specifically from 10 to 15 hpf for the development of *agr2*^*+*^ EMCs.

### Jagged-mediated activation of Notch signaling is required for the differentiation of *agr2*^*+*^ EMCs

**Fig 3:**
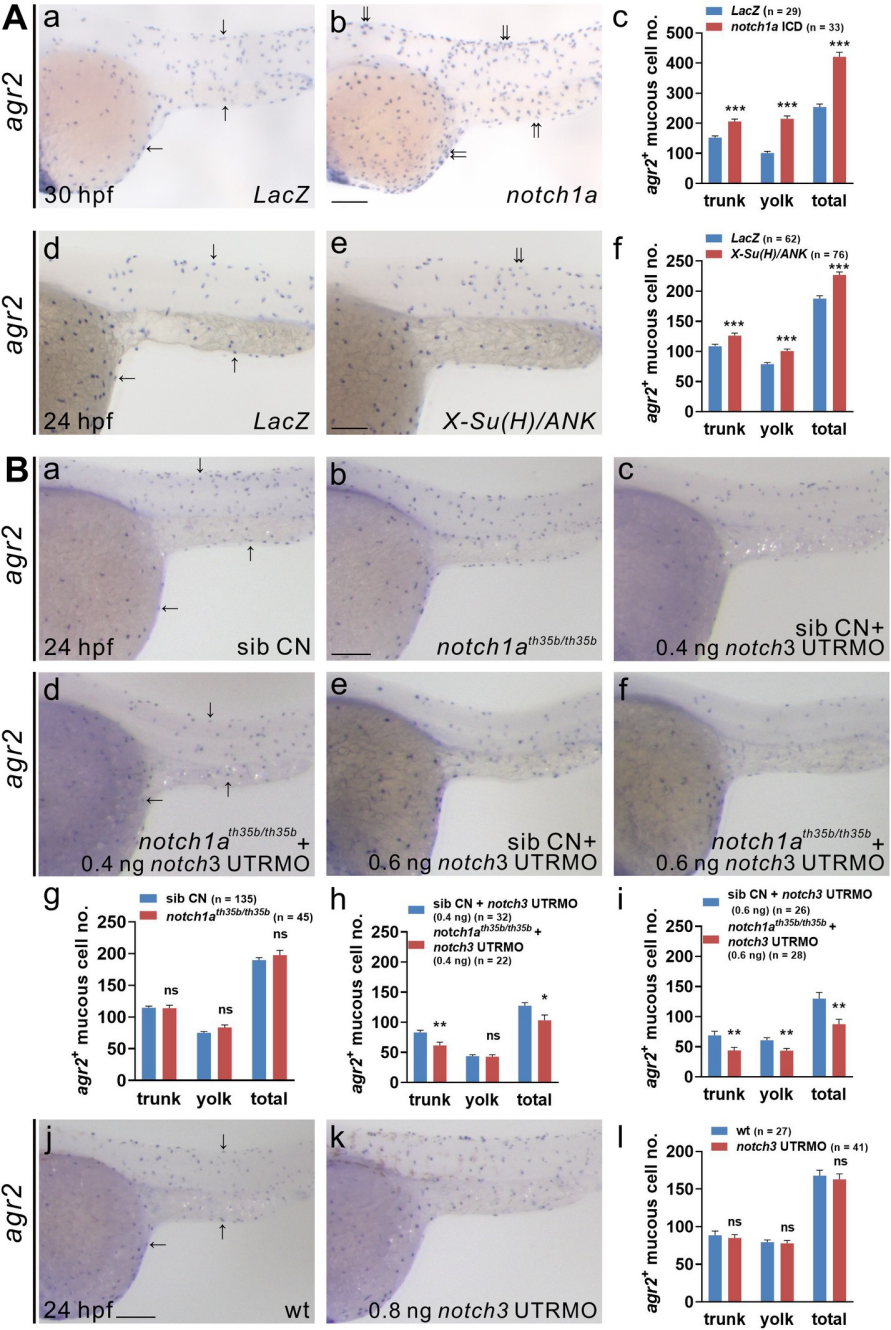
Notch1a and Notch3 receptors participate in the differentiation of *agr2*^*+*^ epidermal mucous cells. **(A).** *notch1a* or *X-Su(H)-ANK-*overexpressing embryos display significant increases in *agr2*^*+*^ EMC numbers in the trunks and yolks compared to *LacZ*-overexpressing embryos at 30 or 24 hpf. **(B).** Similar *agr2*^*+*^ EMC numbers in the trunks and yolks were detected in *notch1a* homozygous mutants or *notch3* morphants compared to sibling wild-type or wild-type embryos at 24 hpf. Substantial reductions in *agr2*^*+*^ EMC numbers in the trunks and yolks are detected in *notch1a* homozygous mutants injected with 0.4 or 0.6 ng *notch3* UTRMO compared to sibling wild types injected with the same amount of *notch3* UTRMO at 24 hpf. Arrows, EMCs. Scale bars, 100 μm. Mean ± SEM. Student’s *t*-test. *p<0.05; **p<0.01; ***p<0.001; ns, not significant. Underlying data are available in S1 Data.

Since Notch1a and Notch3 are determinants of cell fate for ionocytes and keratinocytes [14], we evaluated whether overexpression of *notch1a*, *notch3* or a dominant activator of Notch signaling, *X-Su(H)/ANK* [28], affects the development of *agr2*^*+*^ EMCs. The *agr2*^*+*^ EMC numbers were elevated in the trunks and yolks of embryos injected with 200 pg *notch1a* intracellular domain (ICD) but not *LacZ* mRNA at 19 and 30 hpf (Figs 3Aa-3Ac and S3Aa-S3Ac). Similarly, significantly increased *agr2*^*+*^ EMC numbers were observed in the trunks and yolks of embryos injected with 10 pg *X-Su(H)/ANK* mRNA compared to *LacZ-*injected controls at 24 hpf (Fig 3Ad-3Af). Also, the *pvalb8*-positive cell numbers were increased in the trunks and yolks of embryos injected with 200 pg *notch1a* ICD but not *LacZ* mRNA at 24 hpf (S3Ad-S3Af Fig). These data suggested that activation of Notch signaling via overexpression of *notch1a* ICD or *X-Su(H)/ANK* results in the increased trunk and yolk *agr2*^*+*^ EMC numbers. In addition, Notch signaling activation by *notch1a* ICD overexpression promoted increase of *pvalb8*-positive cell numbers in the trunks and yolks.

In contrast, *agr2*^*+*^ EMC number was diminished in embryos injected with various doses (10 to 200 pg) of *notch3* ICD compared to *LacZ-*injected controls at 24 hpf (S3B Fig). Interestingly, this result is similar to a previous *in vitro* finding that mouse Notch3 ICD inhibits Notch1 ICD-induced *HES* expression in a concentration dependent manner [29]. Thus, overexpressed zebrafish Notch3 ICD may also function as a repressor of endogenous Notch1a ICD to reduce the number of *agr2*^*+*^ EMCs.

We next investigated whether deficiency of *notch1a* or *notch3* affects the development of *agr2*^*+*^ EMCs. The numbers of *agr2*^*+*^ EMCs in the trunks and yolks were similar in *notch1a*^*th35b*^ homozygous mutants and sibling wild-type embryos at 24 hpf (Fig 3Ba, 3Bb, and 3Bg). Likewise, comparable trunk and yolk *agr2*^*+*^ EMC numbers were observed in 0.8 ng *notch3* UTRMO- or 2 ng *notch3* SPMO-injected and wild-type embryos at 24 hpf (Figs 3Bj-3Bl and S3C). We next investigated whether the lack of both *notch1a* and *notch3* affects the development of *agr2*^*+*^ EMCs. The *agr2*^*+*^ EMC numbers in the trunks and yolks of *notch1a*^*th35b*^ homozygous mutants injected with suboptimal doses (0.4 ng and 0.6 ng) of *notch3* UTRMO were greatly reduced compared to sibling controls injected with the same amounts of MO (Fig 3Bc-3Bf, 3Bh, and 3Bi). Similar reduction of *agr2*^*+*^ EMC number was observed in the trunks and yolks of *notch1a*^*th35b*^ homozygous mutants injected with suboptimal doses (1 ng and 1.5 ng) of *notch3* SPMO (S3D Fig). These results indicate that both Notch1a and Notch3 are required for the differentiation of *agr2*^*+*^ EMCs.

**Fig 4:**
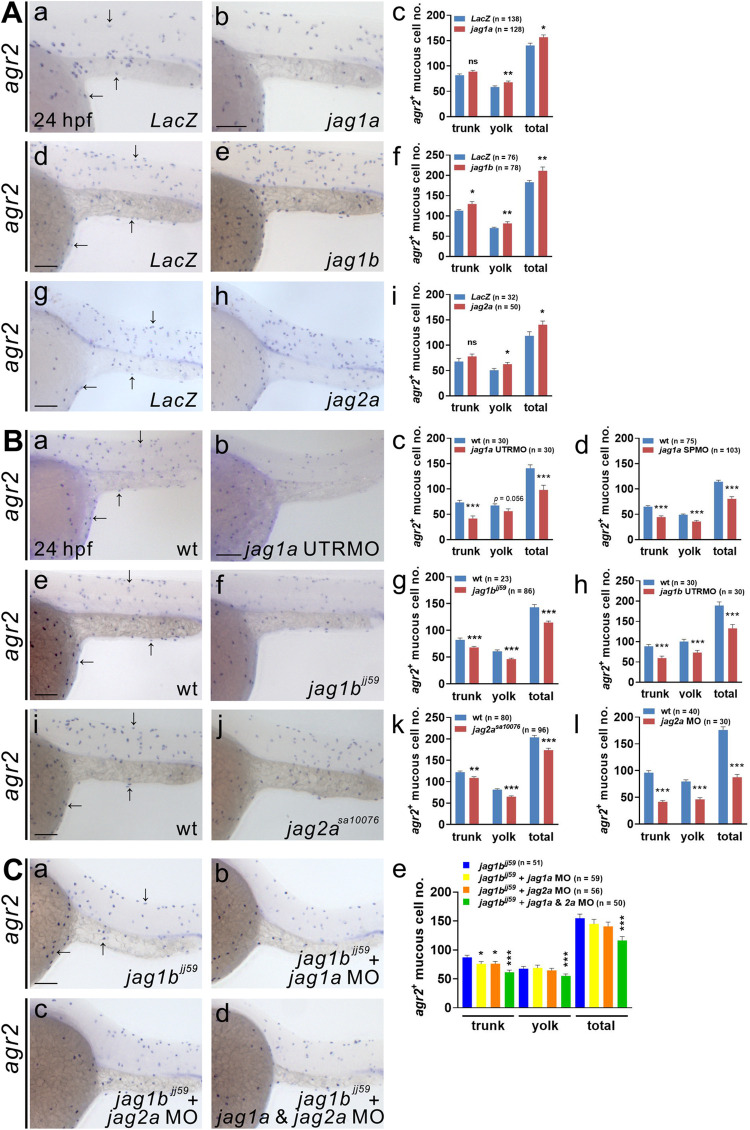
Jagged1a, Jagged1b and Jagged2a ligands participate in the differentiation of *agr2*^*+*^ epidermal mucous cells. **(A).**Significant increases in *agr2*^*+*^ EMC numbers in the trunks and yolks were detected in embryos overexpressing *jag1a*, *jag1b* or *jag2a* mRNA compared to *LacZ* mRNA-overexpressing embryos at 24 hpf. **(B).** Significant reductions in *agr2*^*+*^ EMC numbers in the trunks and yolks were detected in embryos injected with *jag1a* UTRMO, *jag1a* SPMO, *jag1b* UTRMO or *jag2a* MO, as well as *jag1b* and *jag2a* homozygous mutants compared to wild-type embryos at 24 hpf. **(C).** Further reductions in *agr2*^*+*^ EMC numbers in the trunks and yolks were observed in *jag1b* homozygous mutant embryos injected with *jag1a* and *jag2a* MOs compared to those injected with *jag1a* or *jag2a* MO alone or un-injected *jag1b* homozygous mutant embryos at 24 hpf. Arrows, EMCs. Scale bars, 100 μm. Mean ± SEM. Student’s *t*-test. *p<0.05; **p<0.01; ***p<0.001; ns, not significant. Underlying data are available in S1 Data.

Zebrafish has four Jagged ligands, and *jag2a* and *jag2b* are expressed in the epidermis of yolk sac in a punctate pattern at 11 s stage [15], so we next investigated which Jagged ligand is involved in the development of *agr2*^*+*^ EMCs by modulating their expression in zebrafish embryos. Injection of 300 pg *jag1b* but not *LacZ* mRNA substantially increased *agr2*^*+*^ EMC numbers in the trunks and yolks of embryos at 24 hpf (Fig 4Ad-4Af). While injections of 250 pg *jag1a* or 200 pg *jag2a* did not increase *agr2*^*+*^ EMC numbers in the trunk, the *agr2*^*+*^ EMC numbers in the yolk and total EMCs were elevated when compared to *LacZ*-injected embryos at 24 hpf (Fig 4Aa-4Ac, and 4Ag-4Ai).

We then investigated whether deficiency of different Jagged ligands affects the development of *agr2*^*+*^ EMCs. Reduced numbers of trunk and yolk *agr2*^*+*^ EMCs were observed in *jag1b*^*jj59*^ and *jag2a*^*sa10076*^ homozygous mutants (Fig 4Be-4Bg and 4Bi-4Bk) as well as in *jag1b* and *jag2a* morphants (Fig 4Bh and 4Bl) compared to wild-type embryos at 24 hpf. Substantially decreased *agr2*^*+*^ EMC numbers in the trunks and yolks were also observed in embryos injected with *jag1a* UTR MO or *jag1a* SPMO, compared to wild-type embryos (Fig 4Ba-4Bd). In contrast, trunk and yolk *agr2*^*+*^ EMC numbers were similar in *jag1a*^*sa168*^ homozygous mutants and sibling wild-type embryos (S4A Fig). We also evaluated whether the *jag1a*^*sa168*^ mutant is a null allele. We performed DNA sequencing to confirm that *jag1a*^*sa168*^ homozygous mutants harbor a G to T mutation in the gene, which is predicted to encode a truncated Jag1a protein with MNNL, DSL and six EGF domains. Expression levels of *jag1a*, *jag1b*, *jag2a* or *jag2b* were not affected in *jag1a*^*sa168*^ homozygous mutants according to RT-qPCR (S4B Fig). We then knocked down *jag1b* and *jag2a* in wild types and *jag1a*^*sa168*^ mutants. Similar reductions in the numbers of trunk and yolk *agr2*^*+*^ EMCs were identified in *jag1b* and *jag2a* MOs-injected wild-type and *jag1a*^*sa168*^ mutants, compared to un-injected controls at 24 hpf (S4C Fig). Therefore, we suspect that mutated Jag1a protein in *jag1a*^*sa168*^ homozygous mutants still functions normally, at least in terms of *agr2*^*+*^ EMC development. Similarly, no alterations of *agr2*^*+*^ EMC numbers in the trunks and yolks were detected after injection of 500 pg *jag2b* or in *jag2b*^*sa1720*^ heterozygous or homozygous mutants as compared to *LacZ*-injected or sibling wild-type embryos at 24 hpf (S4D Fig).

Low but significant decreases in total *agr2*^*+*^ EMC numbers were detected in *jag1b*^*jj59*^ (19.9%) and *jag2a*^*sa10076*^ (14.6%) homozygous mutant embryos, suggesting that these Jagged ligands have redundant functions. We investigated whether knockdown of *jag1a*, *jag2a* or both genes in *jag1b*^*jj59*^ homozygous mutants could further reduce *agr2*^*+*^ EMC numbers. Compared to un-injected *jag1b*^*jj59*^ homozygous mutants, a 24.8% decrease in the total *agr2*^*+*^ EMC number was detected in *jag1b*^*jj59*^ homozygous mutants injected with both *jag1a* and *jag2a* MOs at 24 hpf (Fig 4C). This result suggests that *jag1a*, *jag1b* and *jag2a* function redundantly during the development of *agr2*^*+*^ EMCs. We also evaluated whether deficiency of *jag1a*, *jag1b* or *jag2a* affects the development of *pvalb8*-positive cells. Similarly, low but significant reductions in trunk and yolk *pvalb8*-positive cell numbers were detected in *jag1a* morphants, *jag1b* morphants and *jag2a* mutants (S4E Fig).

Together, these results demonstrate that Jag1a/1b/2a-mediated activation of Notch1a/3 signaling induces the differentiation of *agr2*^*+*^ EMCs. Likewise, Jagged1a/1b/2a-mediated activation of Notch1a signaling also results in the differentiation of *pvalb8*-positive cells.

### *agr2*^+^ EMCs, ionocytes and keratinocytes are derived from a common non-neural ectodermal precursor

**Fig 5:**
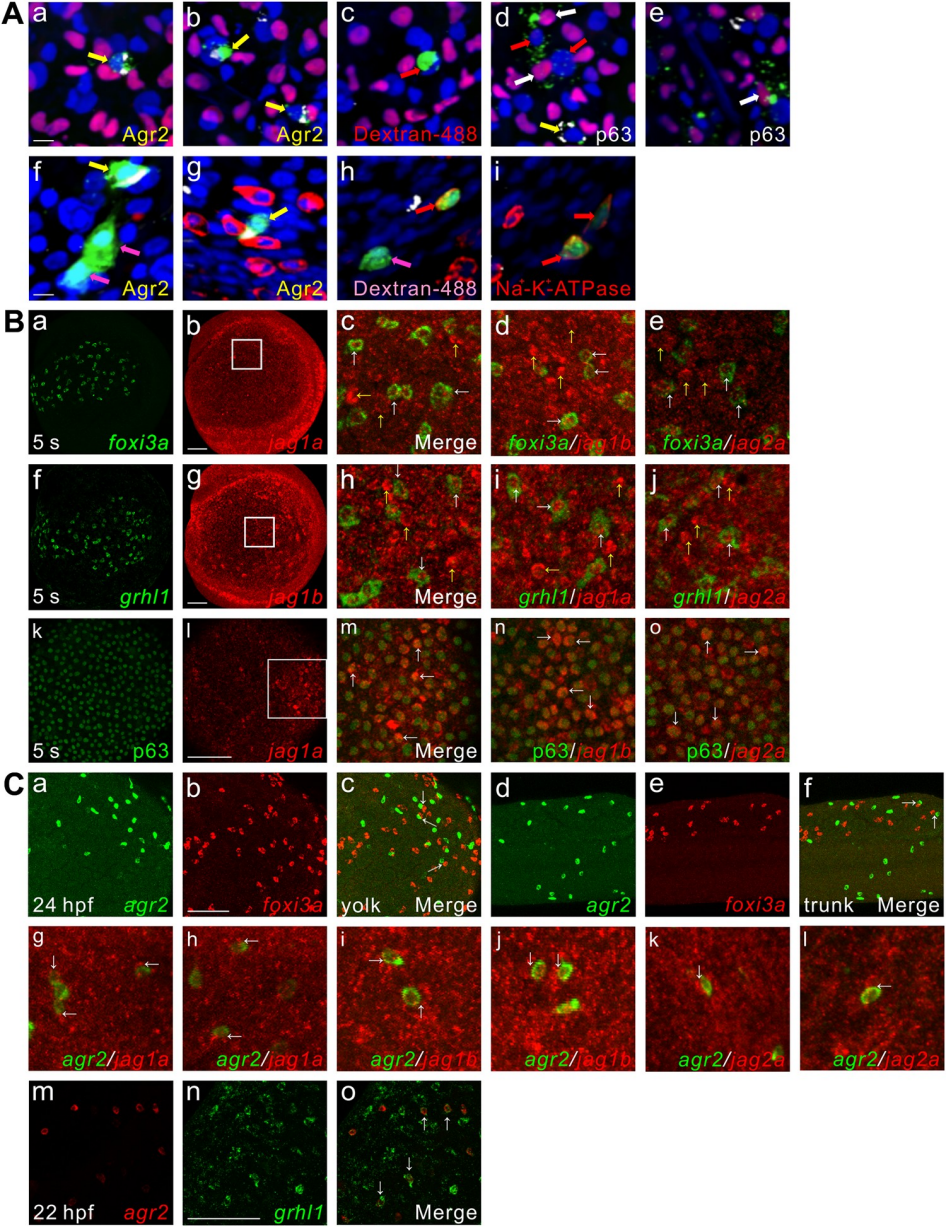
*agr2*^*+*^ EMCs and keratinocytes or ionocytes are derived from common ectodermal precursors, coexpression of *jag1a*/*jag1b*/*jag2a* with *foxi3a*, *grhl1* or p63, *agr2* is expressed next to *foxi3a* or *jag1a*/ *jag1b*/*jag2a* and coexpression of *agr2* and *grhl1*. **(A).** *agr2*^+^ EMCs and keratinocytes or ionocytes are derived from common ectodermal precursors. Yellow arrows (a, b, d, f, g) indicate Agr2^+^ and dextran^+^ (dextran-488) cells. White arrows (d, e) indicate P63^+^ and dextran^+^ cells. Red arrows (c, d) and pink arrows (f, h) indicate only dextran^+^ cells. Red arrows (h, i) indicate Na^+^-K^+^-ATPase^+^ and dextran^+^ cells. Scale bars, 10 μm. **(B).** Coexpression of *jag1a*/*jag1b*/*jag2a* with *foxi3a*, *grhl1* or p63 detected at 5 s. White square boxes indicate enlarged area. White arrows indicate examples of colocalized cells. Yellow arrows indicate example of *jag1a*-, *jag1b*- or *jag2a*-expressing cells. Images (k-o) were derived from 3 to 4 Z-stacks. **(C).** Some *foxi3a*^+^ cells locate next to *agr2*^*+*^ EMCs in the trunks and yolks of embryos at 24 hpf. Some *jag1a-*, *jag1b*- or *jag2a*-expressing cells were located next to *agr2*^*+*^ EMCs in the epidermis of embryos at 22 hpf. Coexpression of *grhl1* and *agr2* was observed in embryos at 22 hpf. White arrows (c, f, g-l) indicate examples of *agr2*^*+*^ EMCs located adjacent to *foxi3a*^+^ or *jag1a-*, *jag1b*- or *jag2a*-expressing cells. White arrows (o) indicate examples of colocalized cells. Scale bars, 100 μm. Underlying data are available in S1 Data.

Previous studies showed that keratinocytes and ionocytes are derived from common precursors from ventral ectoderm [15]. To investigate whether *agr2*^*+*^ EMCs, kerationocyte and ionocytes have the same embryonic origin, we conducted cell-lineage experiments. We transplanted single ventral cells from embryos that had been injected with fluorescent dextran at the 1-cell stage homotypically into unlabeled recipient embryos at shield stage. Chimeric embryos were fixed at 24 hpf and stained with anti-p63 antibody (labels keratinocytes) and anti-salmon Agr2 antibody, or the embryos were stained with anti-Na^+^-K^+^-ATPase antibody and anti- salmon Agr2 antibody (Fig 5A). We identified one embryo (1/7) in which the clones of labeled descendants comprised three p63-positive cells, four Agr2-positive cells and three Agr2/P63-negative cells (Fig 5Aa-5Ae). These results indicate that *agr2*^*+*^ EMCs and keratinocytes are indeed derived from common ectodermal precursors. Similarly, we identified one embryo (1/10) in which the clones of labeled cells consisted of two Agr2-positive cells, three Na^+^-K^+^-ATPase-positive cells, and three Agr2/Na^+^-K^+^-ATPase-negative cells (Fig 5Af-5Ai). These results demonstrate that *agr2*^*+*^ EMCs and ionocytes are also derived from common ectodermal precursors.

Since *jag1a*, *jag1b* and *jag2a* are required for the development of *agr2*^*+*^ EMCs (Fig 4) and *jag2b* was shown to be co-expressed with *foxi3a* and *foxi3b* in ionocyte progenitors at 11 s stage [15], we further conducted double *in situ* hybridization for *foxi3a* or *grhl1* with *jag1a*, *jag1b* or *jag2a*, as well as immunofluorescence staining of p63 with fluorescence *in situ* hybridization for *jag1a*, *jag1b* or *jag2a* at 5 s stage (Fig 5B). *jag1a* exhibited weak expression in a punctate pattern in the epidermis at 5 s (Fig 5Bb), while *jag1b* and *jag2a* showed prominent puncatate expression in the epidermis at 5 s and 10 s (Figs 5Bg and S5Cb, S5Cg, S5Ch). Consistent with previous findings, co-expression of *foxi3a* with *jag1a*, *jag1b* or *jag2a* was identified in the majority of ionocyte progenitors at 5 s. Many *jag1a*^*+*^*/foxi3a*^*-*^ cells, *jag1b*^*+*^/*foxi3a*^*-*^ cells or *jag2a*^*+*^/*foxi3a*^*-*^ cells were also identified at this stage (Fig 5Bc-5Be). Similarly, co-expression of *grhl1* with *jag1b*, *jag1a* or *jag2a* was identified in the majority of non-keratinocyte epidermal cells at 5 s. Many *jag1b*^*+*^*/grhl1*^*-*^ cells, *jag1a*^*+*^/*grhl1*^*-*^ cells or *jag2a*^*+*^/*grhl1*^*-*^ cells were also identified at this stage (Fig 5Bh-5Bj). A previous study showed that *foxi3a* is expressed in a sub-population of p63^+^ epidermal stem cells at bud stage [14], and we also found that *jag1a*, *jag1b* and *jag2a* are expressed in some p63^+^ epidermal stem cells at 5 s stages (Fig 5Bm-5Bo).

We next compared the expression sites of *agr2*^*+*^ EMCs with *foxi3a*, three jagged ligands or *grhl1* by double *in situ* hybridization (Fig 5C). Although some *foxi3a*^+^ ionocytes were localized adjacent to *agr2*^*+*^ EMCs in the yolks and trunks at 24 hpf (Fig 5Cc and 5Cf), we suspect this close proximity may be a random event because knockdown of *foxi3a* did not affect the development of *agr2*^*+*^ EMCs (see S5A Fig). Additionally, we found that approximately 87.5±1.8% *jag1a*^*+*^ (n = 5), 86.0±3.0% *jag1b*^*+*^ (n = 4) and 88.4±4.6% *jag2a*^*+*^ (n = 6) epidermal cells were localized next to *agr2*^*+*^ EMCs at 22 hpf (total yolk *agr2*^*+*^ EMC number was used as the denominator; Fig 5Cg-5Cl), suggesting that *agr2*^*+*^ EMCs may receive Notch signals from neighboring Jagged-expressing cells. Interestingly, co-expression of *agr2* with *grhl1* was observed in the majority of *agr2*^*+*^ EMCs at 22 hpf (Fig 5Cm-5Co); this indicates that *grhl1* is a common marker for all non-keratinocyte epidermal cells including newly-identified *agr2*^*+*^ EMCs and the previously-identified *pvalb8*-positive cells and *foxi3a*^*+*^/*foxi3b*^*+*^ ionocyte progenitors [21].

Co-expression of *foxi3a* and *jag1b*, *jag1a or jag2a* was observed in ionocyte progenitors at 5 s (Fig 5Ba-5Be), suggesting a potential role of ionocytes in the development of *agr2*^*+*^ EMCs. We then investigated whether trunk and yolk *agr2*^*+*^ EMC numbers were affected in *foxi3a* morphants at 24 hpf (S5A Fig). Loss of *atp6v1aa*-positive ionocytes was identified in *foxi3a* morphants compared with wild-types and control embryos injected with *foxi3a* misMO (S5Ae-S5Ag Fig). Unexpectedly, comparable numbers of *agr2*^*+*^ EMCs in the trunk and yolk were observed between *foxi3a* morphants and wild-types. However, low level but significant increases in trunk and total *agr2*^*+*^ EMC numbers were identified in *foxi3a* morphants compared with *foxi3a* misMO-injected control embryos, which may be due to a toxic effect of the *foxi3a* misMO injection (S5Aa-S5Ad Fig).

Since co-expression of *grhl1* and *jag1b*, *jag1a or jag2a* was also observed at 5 s (Fig 5Cm-5Co), we further investigated whether knockdown of *grhl1* affected trunk and yolk *agr2*^*+*^ EMC numbers at 24 hpf (S5B Fig). Similar numbers of *agr2*^*+*^ EMCs in the trunks and yolks were observed among wild-type embryos and those that had been injected with *grhl1* ATGMO or *grhl1* SPMO (S5Ba-d Fig). Consistent with a previous study showing that Grhl1 negatively regulates its own transcription [21], embryos injected with *grhl1* ATGMO or *grhl1* SPMO displayed higher numbers of *grhl1*-positive cells than wild-type controls (S5Be-S5Bg Fig). These results indicate that ionocytes and *grhl1*-positive cells are not the origin of the Jagged signals that promote the development of *agr2*^*+*^ EMCs.

Since Dlc-Notch-mediated lateral inhibition is essential to specify epidermal ionocyte progenitors from epidermal stem cells [14], we wondered whether Dlc participates in the specification of *agr2*^*+*^ EMCs. Our previous study showed that *dlc*^*+*^ ionocyte progenitor numbers reached a maximum at bud stage and were decreased at the 5 s stage [16], so we next conducted double *in situ* hybridization for *dlc* with *jag1b*, *jag1a* or *jag2a* at 5 s and 10 s stages (S5C Fig). The *dlc*^*+*^ ionocyte progenitor number was dramatically reduced at 10 s (S5Cf Fig). Co-expression of *dlc* with *jag2a*, *jag1a* or *jag1b* was observed in some ionocyte progenitors at 5 s and 10 s (S5Ca-S5Ce and S5Cj Fig). Nevertheless, many *jag1b*^*+*^*/dlc*^*-*^ cells, *jag1a*^*+*^*/dlc*^*-*^ cells, or *jag2a*^*+*^*/dlc*^*-*^ cells were identified in the yolk at these two stages (S5Cc-S5Ce, S5Ci, and S5Cj Fig). We then investigated whether deficiency of *dlc* affects trunk and yolk *agr2*^*+*^ EMC numbers (S5D Fig). Comparable numbers of *agr2*^*+*^ EMCs in the trunks and yolks were identified in *dlc*^*tm98*^ mutants and sibling wild-type embryos at 24 hpf (S5Da-S5Dc Fig). Since *dlc*^*tm98*^ mutant is a hypomorphic allele [30], we then investigated the effects of *dlc* morpholino knockown on trunk and yolk *agr2*^*+*^ EMC numbers [31]. Unexpectedly, significant reductions in the trunk and total *agr2*^*+*^ EMC numbers were detected in embryos injected with *dlc* MO compared to wild-type embryos at 24 hpf (S5Dd-f Fig). As in the previous experiments, increases in *foxi3a*^*+*^ ionocyte numbers were observed in *dlc* morphants compared with wild-types at bud stage (S5Dg and S5Dh Fig). We next investigated whether overexpression of *dlc* mRNA affects the development of *agr2*^*+*^ EMCs. Increased numbers of trunk or yolk and total *agr2*^*+*^ EMCs were detected in embryos injected with 92 or 184 pg *dlc* mRNA but not *LacZ* mRNA at 24 hpf (S5Di-S5Dl Fig). However, similar trunk and yolk *pvalb8*-positive cell numbers were observed in embryos injected with 92 or 184 pg *dlc* mRNA or *LacZ* mRNA at 24 hpf (S5Dm-S5Do Fig). We also investigated whether quadruple MO-mediated knockdown of *dlc*, *jag1a*, *jag1b* and *jag2a* could further reduce trunk and yolk *agr2*^*+*^ EMC numbers. Compared to wild-type embryos, a 23.8% decrease in the total *agr2*^*+*^ EMC number was detected in embryos injected with *dlc*, *jag1a*, *jag1b* and *jag2a* MOs (S5Dp-S5Dr Fig). These results suggest that Dlc also participates in the differentiation of *agr2*^*+*^ EMCs but not *pvalb8*-positive cells.

**Fig 6:**
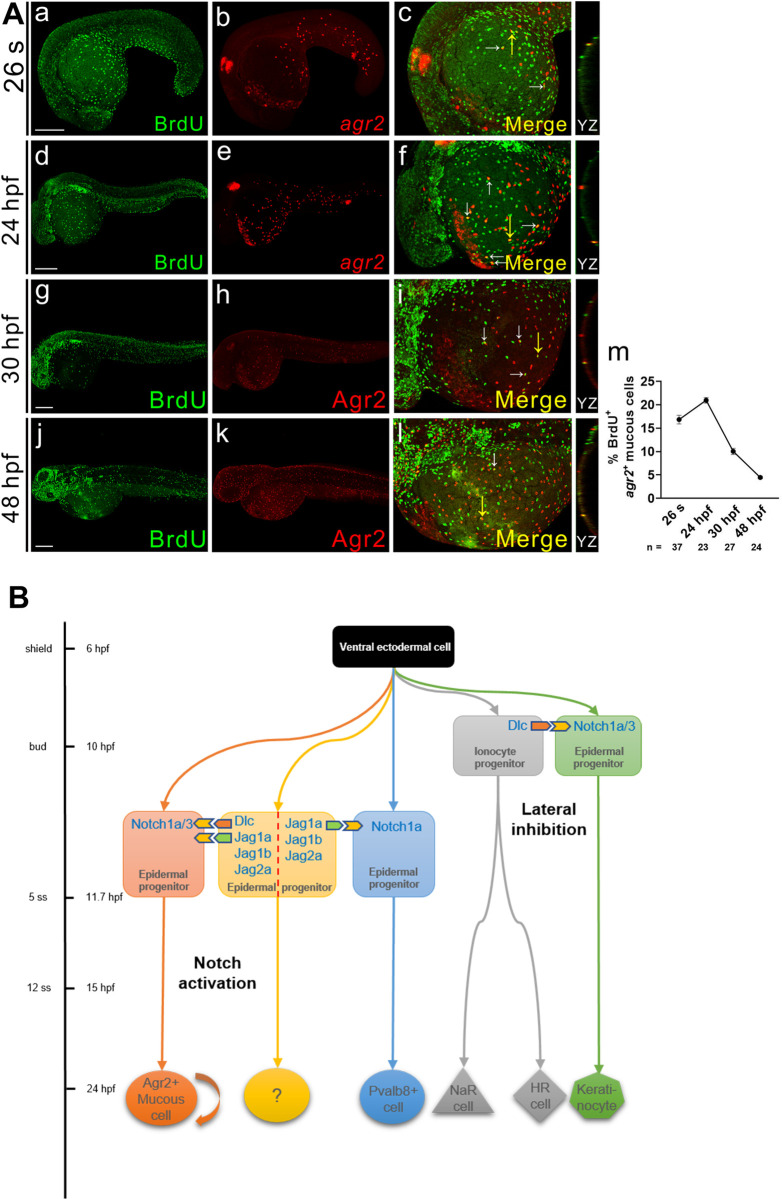
*agr2*^*+*^ EMC population is maintained by proliferation. **(A).** Double immunofluorescence and *in situ* hybridization or double immunofluorescence reveals colocalization of BrdU (green) and *agr2*/Agr2 (red) in the yolks of embryos at 26 s, 24, 30 and 48 hpf. BrdU labeling assay revealed that *agr2*^*+*^ EMCs proliferate after differentiation. The highest proliferation rate was detected at 24 hpf. White arrows indicate BrdU^+^*agr2*^+^ EMCs. Yellow arrows indicate positions of YZ projections of confocal images. Mean ± SEM. Scale bars, 100 μm. **(B)**. A model depicts the timing at which sequential specification of ionocytes/keratinocytes and *agr2*^*+*^ EMCs/*pvalb8*-positive cells occurring in zebrafish embryos. The previously-explored specification of ionocytes and keratinocytes via Dlc-mediated Notch1a/3 lateral inhibition occurs at late gastrulation stage, while the newly-identified induction of *agr2*^+^ EMCs by Dlc and Jag1a/1b/2a-mediated activation of Notch1a/3 signaling and the induction of *pvalb8*-positive cells via Jag1a/Jag1b/Jag2a-mediated activation of Notch1a signaling occur during segmentation stage. Underlying data are available in S1 Data.

Since *agr2*^*+*^ EMC numbers increased with embryonic growth, we wondered how the population is maintained after differentiation. We conducted BrdU labeling and *agr2 in situ* hybridization or Agr2 immunofluorescence on wild-type embryos at 26 s and 24 hpf or 30 and 48 hpf (Fig 6A). A high percentage (16.8%) of BrdU^+^*agr2*^+^ EMCs was observed in the yolk and trunk of 26 s embryos (Fig 6Aa-6Ac and 6Am), and the highest proliferation rate (20.1%) was detected at 24 hpf (Fig 6Ad-6Af and 6Am). Lower percentages (10.2% to 4.4%) of BrdU^+^Agr2^+^ EMCs were observed in the yolk and trunk of embryos at 30 and 48 hpf (Fig 6Ag-6Am).

Together, these results suggest that some p63^+^ epidermal stem cells, but not ionocytes or *grhl1*-positive cells, are the Jagged-expressing cells that activate Notch1a/3 signaling to induce the development of *agr2*^*+*^ EMCs. Then, after differentiation, *agr2*^*+*^ EMCs proliferate to maintain the population.

## Discussion

Among the differentiated epidermal cells in teleosts (keratinocytes, ionocytes and mucous cells), ionocytes and EMCs are evenly dispersed throughout the tissue. The specification of ionocytes begins at late gastrulation stage via Dlc-Notch1a/3-mediated lateral inhibition [14]. In this report, we demonstrate that Dlc/Jag1a/1b/2a-mediated activation of Notch1a/3 signaling directly or indirectly induces the differentiation of EMCs expressing *agr2* during mid-segmentation stages. We also show that the *agr2*^*+*^ EMC population is maintained by proliferation (Fig 6).

We showed that *agr2*^*+*^ EMCs appear initially in the yolk extension at 15 s and in the tail, trunk and yolk sac from 15 s to 22 hpf. Similar to intestinal goblet cells, *agr2*^*+*^ EMCs contain secretory granules and synthesize Muc5.1/5.2 glycoprotein to form a mucus layer (Fig 1). Dramatically reduced numbers of *agr2*^*+*^ EMCs in the yolks and trunks were identified in two *mib* mutants defective in Notch signaling. Furthermore, treatment with a γ-secretase inhibitor also indicated that differentiation of *agr2*^*+*^ EMCs requires Notch signaling specifically from 10 to 15 hpf (Fig 2). We then identified Notch1a and Notch3 receptors and three Jagged ligands (Jag1a, Jag1b and Jag2a) as major components in the differentiation of *agr2*^*+*^ EMCs, according to overexpression, morpholino knockdown and mutant analyses (Fig 3 and Fig 4). Cell lineage experiments further demonstrated that *agr2*^*+*^ EMCs and keratinocytes or ionocytes are derived from common ectodermal precursors (Fig 5).

Dlc and Jag1a/1b/2a are two alternate ligands of Notch signaling and they specify two major cell types in the epidermis via a streamlined Notch-dependent process. The specification of ionocytes by Dlc-Notch1a/3 mediated lateral inhibition occurs early, during late gastrulation stage [14], while Jag1a/1b/2a-mediated Notch1a/3 activation induces differentiation of *agr2*^*+*^ EMCs later, during mid-segmentation stages (Fig 6B). Although Dlc is mainly responsible for the first specification event, knockdown of *dlc* decreased trunk and total *agr2*^*+*^ EMC numbers, and overexpression of *dlc* increased trunk or yolk and total *agr2*^*+*^ EMC numbers (S5D Fig). These results indicate that Dlc also participates in the differentiation of *agr2*^*+*^ EMCs. Interestingly, a 23.8% decrease in the total *agr2*^*+*^ EMC number was detected in embryos injected with four MOs against *dlc*, *jag1a*, *jag1b* and *jag2a*; this level of decrease is similar to that (24.8%) observed in *jag1b*^*jj59*^ homozygous mutants injected with both *jag1a* and *jag2a* MOs (Figs S5D and 4C).This result may be due to that *dlc*^*+*^ ionocyte progenitor cell numbers reached a maximum at bud stage and were diminished at 10 s stage (S5C Fig) [16]. In contrast, epidermal cells expressing *jag1a*, *jag1b* or *jag2a* were identified in embryos at 5 s (Fig 5B), 10 s (S5C Fig), 22 hpf (Fig 5C) and 30 hpf [32]. Although co-expression of *dlc* or *foxi3a* with *jag1a*, *jag1b* or *jag2a* was observed at 5 s stage (Figs 5B and S5C), the identified cells may not participate in the specification of ionocytes because no alterations in numbers of *atp1b1b*^*+*^ or *atp6v1aa*^*+*^ ionocytes were observed in embryos injected with single or quadruple MOs against all four *jag* genes [15]. Expression of various *jag* genes occurs during 5 s to 10 s stage, which is also in line with the conclusion that Notch signaling is required from 10 to 15 hpf for the development of *agr2*^*+*^ EMCs (Fig 2). In addition, epidermal cells expressing *jag1a*, *jag1b or jag2a* were localized nearby *agr2*^*+*^ EMCs at 22 hpf (Fig 5C). These results support the conclusion that the second specification event is initiated by Jag1a/1b/2a ligands.

Although coexpression of three *jag* ligands was identified in ionocyte progenitor and non-keratinocyte markers, knockdowns of *foxi3a* or *grhl1* do not affect the development of *agr2*^*+*^ EMCs (S5A and S5B Fig). Nevertheless, we found coexpression of *jag1a*, *jag1b* and *jag2a* in some p63^+^ epidermal stem cells at 5 s (Fig 5B), suggesting that p63^+^ epidermal stem cells are the Jag1a, Jag1b, and Jag2a presenting cells for *agr2*^*+*^ EMC differentiation. A previous publication reported that expression of human *JAG1* and *JAG2* was upregulated by p63 and a p63-binding site in the *JAG1* gene that interacts with p63 protein was identified *in vivo* [33]. These findings further support the idea that p63^+^ epidermal progenitor cells expressing Jag1a, Jag1b, and Jag2a are responsible for inducing the formation of *agr2*^*+*^ EMCs in neighboring cells. While our model indicates sequential specification of ionocytes/kerationocytes and *agr2*^*+*^ EMCs via Dlc-Notch1a/3-mediated lateral inhibition and Dlc/Jag1a/1b/2a-mediated Notch 1a/3 activation (Fig 6B), a variant of this model could also be proposed. That is, early developed p63^+^ keratinocytes may begin to express Jag1a/1b/2a during bud to 15 hpf and supply Notch1a/3 signal that activates differentiation of neighboring epidermal cells into *agr2*^*+*^ EMCs. However, the cell fates of these *jag1a/1b/2a*-expressing epidermal cells are still unknown (Fig 6B). Single cell RNA seq of △Np63-lineage traced epidermal cells may provide definitive answers in the future.

Additionally, we showed that *agr2*^*+*^ EMCs and *pvalb8*-positive cells may represent two different populations of EMCs (S1 Fig). Significantly reduced trunk and yolk *pvalb8*-positive cell numbers were observed in *mib*^*ta52b*^ and *mib*^*tfi91*^ mutants as well as *jag1a* morphants, *jag1b* morphants and *jag2a* mutants (S2A and S4E Figs), while increased trunk and yolk *pvalb8*-positive cell numbers were identified in *notch1a*-overexpressing, but not *dlc*-overexpressing embryos (S3A and S5D Figs). Unlike *pvalb8*-positive cell numbers, which were negatively regulated by Foxi3a [21], the *agr2*^*+*^ EMC numbers in *foxi3a* morphants and wild-type embryos were similar at 24 hpf (S5A Fig). Such differential regulation of Foxi3a on numbers of *pvalb8*-positive cells and *agr2*^*+*^ EMCs remains to be studied in the future. Nevertheless, our results suggest that Jag1a/1b/2a-mediated Notch signaling activation might participate in the development of mucous cell types other than *agr2*^*+*^ EMCs, such as *pvalb8*-expressing putative EMCs.

Although several major features of teleost and mammalian epithelia are different, both function to protect organisms from environmental challenges. Moreover, the activation of Notch1 signaling by Delta-1 or Jagged-1 has been implicated in early and late events of keratinocyte differentiation within mammalian epidermis [34,35]. Within the stratified epithelium, Notch signaling induces terminal differentiation of spinous cells in a Hes1-independent manner; Notch signaling also promotes *Ascl2* transcription to induce granular differentiation, which is repressed by Hes1 protein [36]. Similarly, we showed that activation of Notch1a/3 signaling by Dlc/Jag1a/1b/2a promote the differentiation of *agr2*^*+*^ EMCs in zebrafish embryos. Therefore, Notch signaling appears to exert an essential and conserved role in the differentiation of epithelial cells, such as EMCs in teleosts and keratinocytes in terrestrial vertebrates.

After induction of *agr2*^*+*^ EMCs by Delta/Jagged-mediated activation of Notch1a/3, the *agr2*^*+*^ EMCs population is maintained by proliferation (Fig 6A). High proliferation rates (16.8–20%) were detected in 26 s and 24 hpf embryos, while a lower (4.4%) proliferation rate was observed at 48 hpf. Likewise, after specification by Dlc-Notch1a/3 lateral inhibition during late gastrulation and early somite stages, ionocytes also maintain their population by proliferation; a similarly low (3%) proliferation rate was observed in NaR cells at 120 hpf [37]. In contrast, mouse Notch1 coordinates keratinocyte growth arrest and differentiation by induction of p21^WAF1/Cip1^ expression and early differentiation markers (keratin 14, integrin β1 and β4) [8].

Although EMCs and intestinal goblet cells both express *agr2* and serve primarily as secretory cells, their differentiation and regulation are quite different. In zebrafish intestine, Delta-Notch signaling controls the decision between secretory and absorptive cell fates [38]. Most intestinal cells adopt a secretory cell fate in DeltaD-defective *aei* mutants and *mib* mutant fish embryos. On the contrary, we observed reduced *agr2*^*+*^ EMC numbers in various mutants or morphants defective in Dlc and Jagged ligands (*jag1a/1b/2a*) or Notch receptors (*notch1a/3*), and *mib* mutants (Figs 2, 3, 4, and S5). We suspect that the choice between cell fate specification through Delta-Notch lateral inhibition or Delta/Jagged-induced Notch signaling may determine the distribution patterns and abundances of goblet and mucous cells in the intestine or epidermis. Overall, our findings reveal that the differentiation of *agr2*^*+*^ EMCs is controlled by Delta/Jagged ligand induction of Notch signaling. This result is consistent with a recent publication that showed Notch signaling induces a secretory cell fate change in epidermal multiciliated cells of *Xenopus* embryos [39]. Thus, our results suggest that the specification of three major cell types in the epidermis depends on temporally distinct Notch-dependent signaling events, and this consistent involvement of Notch signaling in epidermal cell development may be common to many vertebrate species.

## Materials and methods

### Ethics statement

All animal procedures were approved by the Academia Sinica Institutional Animal Care & Use Committee (AS IACUC; Protocol ID: 15-12-918 and 18-12-1250) or by the Institutional Animal Care and Use Committee, NHRI, Taiwan (NHRI-IACUC-106063-A, NHRI-IACUC-107094-A and NHRI-IACUC-109036-A). All animal experiments were carried out in accordance with the approved protocols.

### Zebrafish strains and breeding conditions

Adult zebrafish were maintained in a high-density self-circulation system (Aqua Blue) or in 20-liter aquaria supplied with aerated filtered fresh water, located in a room with a 14/10-h light/dark photoperiod. Embryos were incubated at 28.5°C or 25°C depending on the stage at which they were harvested. Stage determination was based on described criteria [40]. Various mutants, transgenic lines and wild-types used in this study were: *dlc*^*tm98/+*^, *mib*^*ta52b/+*^, *mib*^*tfi91/+*^, *jag1a*^*sa168/+*^, *jag1b*^*jj59/+*^, *jag2a*^*sa10076/+*^, *jag2b*^*sa1720/+*^, *notch1a*^*th35b/+*^, *Tg(-6*.*0k agr2*:*EGFP)*^*as11Tg*^, AB wild types, and ASAB wild types.

### Morpholino knockdown, capped mRNA and probe synthesis and mRNA overexpression

Various morpholio oligonucleotides (MOs) from Gene Tools used in this study were as follows: *dlc* MO: 5’-CGATAGCAGACTGTGAGAGTAGTCC -3’, 4.9 ng [31]; *foxi3a* ATGMO: 5’-AGACTGTGGAACAAATGATGTCATG -3’, 4.3 ng [14]; *foxi3a* misMO: 5’-AGA*G*TGTG*C*AAGAAAT*C*ATGT*G*ATG-3’, 4.3 ng (mismatched sequence is italized) [14]; *grhl1* ATGMO: 5’-GTGACATCTCTTATGGTCGAACTGG-3’, 3 ng [21]; *grhl1* SPMO: 5’-CTTTGATGAGAGCTTCACCTTTTGT-3’, 3 ng [21]; *jag1a* UTRMO: 5’- CGGTTTGTCTGTCTGTGTGTCTGTC-3’, 0.5 ng [41]; *jag1a* SPMO: 5’- ATAAAAGATACTCACTGCCAGTGCT-3’, 1.0 ng; *jag1b* UTRMO: 5’- TCACGGCTCTAATGTACTCCCCGAT-3’, 1.2 ng [42]; *jag2a* MO: 5’- CTCCAAAATAGTTATGCATGACTCC-3’, 4 ng (start codon is underlined); *notch3* UTRMO: 5’-ACATCCTTTAAGAAATGAATCGGCG-3’, 0.8 ng [43]; *notch3* SPMO: 5’- AAGGATCAGTCATCTTACCTTCGCT-3’, 2 ng [43] Various MOs were diluted in Danieau solution and individually injected into the cytoplasm of one- or two-cell zygotes.

Capped full-length *dlc*, *jag1a*, *jag1b*, *jag2a*, *jag2b*, *LacZ*, *notch1a* ICD, *notch3* ICD, and *X-Su(H)/ANK* mRNA were synthesized using NotI-linearied PCS2^+^-MT-*dlc* plasmid, NotI-linearized pCS2^+^-MT-*jag1a* plasmid, NotI-linearized pCS2^+^-MT-*jag1b* plasmid, NotI-linearized pCS2^+^-*jag2a* plasmid, SacII-linearized pCS2^+^-*jag2b* plasmid, NotI-linearized pcDNA3.1/Myc-His(+)/*LacZ* plasmid, NotI-linearized pCS2^+^-MT-*notch1a* ICD plasmid, NotI-linearized pCS3^+^-MT-*notch3* ICD plasmid, and NotI-linearized pCS2^+^-*X-Su(H)-ANK* plasmid as templates with the mMESSAGE mMACHINE SP6 or T7 transcription kit (Invitrogen). Digoxigenin (DIG)- or fluorescein-labeled RNA probes were generated using respective linearized constructs as templates and DIG or fluorescein RNA labeling kit with SP6 or T7 RNA polymerase (SP6, Roche; T7, Invitrogen). Restriction enzymes used for linearization and RNA polymerase for probe synthesis were: *agr2* (NcoI/SP6), *atp6v1aa* (SacII/SP6), *dlc* (NcoI/SP6), *egfp* (HindIII/SP6), *grhl1* (SalI/T7), *jag1a* (NcoI/SP6), *jag1b* (SalI/T7), *jag2a* (SalI/T7), *mucin 5*.*1*(NcoI/SP6), *mucin 5*.*2* (NcoI/SP6) and *pvalb8* (SalI/T7). Overexpression experiments were conducted by microinjecting 92 or 184 pg *dlc*, 250 pg *jag1a*, 300 pg *jag1b*, 200 pg *jag2a*, 500 pg *jag2b*, 200 pg *notch1a* ICD, *notch3* ICD (10, 50, 100, 200 pg) or 10 pg *X-Su(H)-ANK* into the cytoplasm of one- or two-cell zygotes.

### MK-0752 treatment

Working solutions were prepared from 10 mM MK-0752 (Selleckchem) stock solution and 100% DMSO (Invitrogen) in 1x E3 medium (5 mM NaCl, 0.17 mM KCl, 0.33 mM CaCl_2_, 0.33 mM MgSO_4_). Embryos (20 embryos/mL) were incubated with 100 μM MK-0752 or 1% DMSO from bud to 17 or 20 hpf at 28.5°C, followed by 1x E3 medium washes and maintenance at 28.5°C. Embryos were maintained until 24 hpf, at which point they were fixed with 4% paraformaldehyde (PFA) in 1x PBS at 4°C overnight. Embryos were also incubated with 100 μM MK-0752 or 1% DMSO from bud to 24 hpf at 28.5°C before fixation. Embryos were incubated with 50 μM MK-0752 or 0.5% DMSO from bud to 13 or 14 hpf, or from 11 to 14 or 15 hpf, followed by 1x E3 medium washes and maintained at 28.5°C until 24 hpf stage for fixation. Embryos were also incubated with 75 μM MK-0752 or 0.75% DMSO from bud to 15, 16 or 17 hpf, or from 12 to 17 hpf at 28.5°C, followed by 1x E3 medium washes; embryos were maintained at 28.5°C until fixation at the 24 hpf stage.

### BrdU incorporation

Embryos at 24 s, 23 hpf, 29 hpf or 47 hpf were treated with egg water containing 10 mM BrdU and 15% DMSO in 2 mL tube for 20 min on ice. Samples were then transferred to a 9-cm petri dish and washed with egg water five times, then incubated for 40 min at 28.5°C before fixation at 26 s, 24 hpf, 30 hpf and 48 hpf with 4% PFA in 1x PBS at 4°C overnight. After several PBST washes, embryos were dehydrated with 25% methanol/75% PBST, 50% methanol/50% PBST, 75% methanol/25% PBST and 100% methanol for 5 min, three times, and stored in 100% methanol at -20°C.

### Whole-mount *in situ* hybridization

Dechorionated embryos at 24 hpf were fixed with freshly prepared 4% PFA in 1x PBS at 4°C overnight. Embryos were washed with diethyl pyrocarbonate (DEPC)-treated 1x PBS containing 0.1% Tween 20 (PBST) for 5 min at room temperature (RT), three times, followed by dehydration with 25% methanol/75% DEPC-PBST, 50% methanol/50% DEPC-PBST, 75% methanol/25% DEPC-PBST and 100% methanol and storage in 100% methanol at -20°C. After rehydration with a methanol/DEPC-PBST series and DEPC-PBST washes, embryos were digested with 10 μg/mL proteinase K (Roche) in DEPC-PBST for 3 min at RT, followed by DEPC-PBST washes and re-fixation with 4% PFA in 1x PBS for 20 min at RT. After several DEPC-PBST washes, embryos were incubated in hybridization buffer (50% formamide, 5x SSC, 0.1% Tween-20, 500 μg/mL yeast tRNA, 50 μg/mL heparin adjusted to pH 5.2 with 2 M citric acid) at 65°C for 3 h before incubation in probe solution (150 ng RNA probe per 300 μL hybridization buffer) at 65°C overnight for hybridization. Embryos were washed at 65°C with pre-heated 25%, 50%, 75% Hyb^-^ buffer (50% formamide, 5x SSC, 0.1% Tween-20) in 2x SSC for 10 min each, 2x SSC for 10 min then 1 h and 0.2x SSC containing 0.1% Tween-20 for 30 min four times at 70°C. Embryos were then incubated in 1% blocking reagent (Roche) for 3 h at RT before incubation with pre-absorbed anti-digoxygenin (DIG)-alkaline phosphatase antibody (1:4000) in 0.5% blocking solution at 4°C overnight. After several PBST washes, embryos were equilibrated in freshly made staining buffer (0.1 M Tris-HCl pH 9.5, 50 mM MgCl_2_, 0.1 M NaCl, 0.1% Tween-20) for 5 min three times and later stained with NBT/BCIP-containing staining buffer (3.5 μL of 100 mg/mL NBT in 70% dimethylformamide and 3.5 μL of 50 mg/mL X-phosphate in dimethylformamide added to 1 mL staining buffer) until the color signal was clearly apparent. Embryos were rinsed with PBST, washed in 100% methanol for 20 min. After several PBST washes, embryos were stored in 4% PFA in 1x PBS at 4°C before samples were photographed.

### Double immunofluorescence for Agr2 and EGFP

Dechorinated 48 hpf *Tg(-6*.*0k agr2*:*EGFP)* embryos were fixed with 4% PFA in 1x PBS at 4°C overnight. After four washes with PBST for 5 min at RT, embryos were dehydrated with 25% methanol/75% PBST, 50% methanol/50% PBST, 75% methanol/25% PBST and 100% methanol for 5 min, three times, and stored in 100% methanol at -20°C. Embryos were treated with acetone for 15 min at -20°C before being rehydrated through 75% methanol/25% PBST, 50% methanol/50% PBST, 25% methanol/75% PBST and PBST four times for 5 min each. Embryos were treated with 150 mM Tris-HCl, pH 9.5 for 15 min at 70°C, followed by re-fixation with 4% PFA in 1x PBS for 5–10 min at RT. After four PBST washes for 5 min each, embryos were blocked in PBST containing 1% blocking reagent for 1.5 h at RT. Embryos were then treated with anti-salmon Agr2 antibody (1:200) and anti-GFP antibody (1:400, Sigma-Aldrich) in PBST containing 1% blocking reagent at 4°C overnight. After PBST washes for 10 min, six times, embryos were blocked with 1% blocking reagent for 1 h at RT followed by incubation with anti-mouse Alexa Fluor 488 antibody (1:200, Invitrogen) and anti-rabbit Alexa Fluor 568 antibody (1:200, Invitrogen). After six PBST washes for 10 min each, embryos were stored in 4% PFA in 1x PBS at 4°C before being photographed.

### Double fluorescence *in situ* hybridization for *egfp* and *muc5*.*1/5*.*2*, *egfp* and *pvalb8*, *agr2* and *foxi3a* or *agr2* and *grhl1*

Dechorinated *Tg(-6*.*0k agr2*:*EGFP)* embryos at 24 or 48 hpf and dechorinated wild-type embryos at 22 or 24 hpf were fixed with 4% PFA in 1x PBS at 4°C overnight. After four DEPC-PBST washes for 5 min each, embryos were dehydrated through 25% methanol/75% DEPC-PBST, 50% methanol/50% DEPC-PBST, 75% methanol/25% DEPC-PBST and 100% methanol and stored in 100% methanol at -20°C. After rehydration with a methanol/DEPC-PBST series and DEPC-PBST washes, embryos were digested with 10 μg/mL proteinase K in DEPC-PBST (1–3 min for 22 hpf, 3 min for 24 hpf and 20 min for 48 hpf) at RT, followed by DEPC-PBST washes and re-fixation with 4% PFA in 1x PBS for 20 min at RT. After several DEPC-PBST washes, embryos were incubated in hybridization buffer (50% formamide, 5x SSC, 0.1% Tween-20, 500 μg/mL yeast tRNA, 50 μg/mL heparin adjusted to pH 5.2 with 2 M citric acid) at 65°C for 1 h before incubation in probe solution (300 ng each for *muc5*.*1* and *muc5*.*2*, 300 ng *pvalb8* and 150 ng *eGFP* RNA probes, 150 ng *agr2* and 150 ng *foxi3a* or 150 ng *grhl1* in 300 μL hybridization buffer) at 65°C overnight for hybridization. Embryos were washed with pre-heated 75%, 50%, 25% Hyb^-^ buffer in 2x SSC for 10 min each, 2x SSC for 30 min, two times, and 0.2x SSC for 30 min four times at 70°C. After two PBST washes for 5 min each, embryos were blocked in 1% blocking reagent in PBST for 1.5 h at RT, followed by incubation with anti-DIG- horse-radish peroxidase (POD) antibody (1:600) in 0.5% blocking reagent at 4°C overnight. After six PBST washes for 15 min each, embryos were incubated for 45 to 60 min at RT with TSA plus Cy3-tyramide substrate (1:100, Akoya Biosciences) diluted in amplification buffer. Embryos then washed in PBST, 25% PBST/75% methanol, 50% PBST/50% methanol, 25% PBST/75% methanol and 100% methanol for 10 min each at RT, followed by inactivation of POD with 1% H_2_O_2_ in 100% methanol for 30 min at RT. Embryos were then blocked with 1% blocking reagent in PBST for 1 h at RT, followed by incubation with anti-fluorescein-POD antibody (1:600) in 0.5% blocking reagent at 4°C overnight. Embryos were washed with PBST for 15 min six times, followed by incubation in TSA plus FITC-tyramide substrate (1:100) diluted in amplification buffer for 45 to 60 min at RT. Embryos were washed with PBST for 10 min four times and stored in 4% PFA in 1x PBS at 4°C.

### Double fluorescence *in situ* hybridization for *jag1a*/*jag1b*/*jag2a* and *foxi3a*, *grhl1*, *dlc* or *agr2*

Dechorinated wild-type embryos at 5 s, 10 s or 22 hpf were fixed with 4% PFA in 1x PBS at 4°C overnight. After four DEPC-PBST washes for 5 min each, embryos were dehydrated through 25% methanol/75% DEPC-PBST, 50% methanol/50% DEPC-PBST, 75% methanol/25% DEPC-PBST and 100% methanol and stored in 100% methanol at -20°C. After rehydration with a methanol/DEPC-PBST series and DEPC-PBST washes, embryos were digested with 10 μg/mL proteinase K in DEPC-PBST for 1 min at RT, followed by DEPC-PBST washes and re-fixation with 4% PFA in 1x PBS for 20 min at RT. After several DEPC-PBST washes, embryos were incubated in hybridization buffer (50% formamide, 5x SSC, 0.1% Tween-20, 500 μg/mL yeast tRNA, 50 μg/mL heparin adjusted to pH 5.2 with 2 M citric acid) at 65°C for 4 h before incubation in probe solution (200 ng each for *jag1a*, *jag1b* and *jag2a* and 150 ng *dlc*, 150 ng *foxi3a* or 150 ng *agr2* in 300 μL hybridization buffer) at 65°C overnight for hybridization. Embryos were washed with pre-heated 75%, 50%, 25% Hyb^-^ buffer in 2x SSC for 10 min each, 2x SSC for 30 min, two times, and 0.2x SSC for 30 min four times at 65°C. After two PBST washes for 5 min each, embryos were blocked in 1% blocking reagent in 1x PBST for 3 h at RT, followed by incubation with anti-DIG-POD antibody (1:600) in 0.5% blocking reagent at 4°C overnight. After six to eight PBST washes for 15 min each, embryos were incubated for 40 min at RT with TSA plus Cy3-tyramide substrate (1:100, Akoya Biosciences) diluted in amplification buffer. Embryos were washed for 10 min three times in PBST and incubated in 100 mM glycin pH 2.2 for 10 min at RT. Embryos were washed for 10 min each in 50% methanol/50% PBST and 100% methanol at RT, followed by PBST washes for 10 min two times. Embryos were blocked in 1% blocking reagent in PBST for 1 h at RT, followed by incubation with anti-fluorescein-POD antibody (1:600) in 0.5% blocking reagent at 4°C overnight. Embryos were washed with PBST for 15 min six to eight times, followed by incubation in TSA plus FITC-tyramide substrate (1:100) diluted in amplification buffer for 40 min at RT. Embryos were washed with PBST for 10 min four times and stored in 4% PFA in 1x PBS at 4°C.

### Imunofluorescence for p63 and fluorescence *in situ* hybridization for *jag1a*, *jag1b* or *jag2a*

Dechorinated wild-type embryos at 5 s were fixed with 4% PFA in 1x PBS at 4°C overnight. After four DEPC-PBST washes for 5 min each, embryos were dehydrated through 25% methanol/75% DEPC-PBST, 50% methanol/50% DEPC-PBST, 75% methanol/25% DEPC-PBST and 100% methanol and stored in 100% methanol at -20°C. After rehydration with a methanol/DEPC-PBST series and DEPC-PBST washes, embryos were digested with 10 μg/mL proteinase K in DEPC-PBST for 1 min at RT, followed by DEPC-PBST washes and re-fixation with 4% PFA in 1x PBS for 20 min at RT. After several DEPC-PBST washes, embryos were incubated in hybridization buffer (50% formamide, 5x SSC, 0.1% Tween-20, 500 μg/mL yeast tRNA, 50 μg/mL heparin adjusted to pH 5.2 with 2 M citric acid) at 65°C for 4 h before incubation in probe solution (200 ng each for *jag1a*, *jag1b* and *jag2a* in 300 μL hybridization buffer) at 65°C overnight for hybridization. Embryos were washed with pre-heated 75%, 50%, 25% Hyb^-^ buffer in 2x SSC for 10 min each, 2x SSC for 30 min, two times, and 0.2x SSC for 30 min four times at 65°C. After two PBST washes for 5 min each, embryos were blocked in 1% blocking reagent in PBST for 3 h at RT, followed by incubation with anti-DIG-POD antibody (1:600) in 0.5% blocking reagent at 4°C overnight. After six to eight PBST washes for 15 min each, embryos were incubated for 40 min at RT with TSA plus Cy3-tyramide substrate (1:100, Akoya Biosciences) diluted in amplification buffer. Embryos were washed for 10 min three times in PBST, followed by incubation in 100 mM glycine pH 2.2 for 10 min. Embryos were washed for 10 min each in 50% methanol/50% PBST and 100% methanol at RT, followed by PBST washes for 10 min two times. Embryos were treated with 150 mM Tris-HCl, pH 9.5 for 15 min at 70°C, followed by re-fixation with 4% PFA in 1x PBS for 5–10 min at RT. After four PBST washes for 5 min each, embryos were blocked in PBST containing 0.5% blocking reagent for 1 h at RT. Embryos then treated with anti-p63 antibody (1:50, Abcam) in PBST containing 0.5% blocking reagent at 4°C overnight. After PBST washes for 10 min, six times, embryos were blocked with 1% blocking reagent for 1 h at RT followed by incubation with anti-mouse Alexa Fluor 488 antibody (1:200, Invitrogen). After PBST washes for 10 min, six times, embryos were stored in 4% PFA in 1x PBS at 4°C.

### Lineage tracing experiments

The donor embryos were injected with 5% dextran-Alexa Fluor 488 (anionic, fixable, 10 kDa) in 0.1 M KCl at the 1-cell stage. To obtain single cells for transplantation, cells from the ventral ectoderm at shield stage were loaded into a transplant needle (10 psi) and released on a 1.5% agarose plate containing PBS. Single cells were then loaded into a needle and transferred into the ventral ectoderm region of host embryos. Dechorinated 24 hpf recipients were fixed with 4% PFA at 4°C overnight. For Agr2^+^ cells and keratinocytes, fixed embryos were washed with 1x PBS containing 0.3% triton X-100 (PBT) for 5 min three times at RT. After washing with PBS for 5 min twice, embryos were treated with 10 μg/mL proteinase K for 5 min at 28°C. Embryos were then washed twice with PBT for 5 min each, and further fixed with 4% PFA for 20 min at RT. After washing with PBT for 5 min three times, embryos were blocked with 4% BSA in PBT for 1 h at RT. Embryos were incubated with rabbit anti-salmon Agr2 antibody (1:200) and mouse anti-human P63 antibody (1:200; 4A4, Abcam) in 4% BSA at 4°C overnight. After washing with PBT for 15 min four times, embryos were incubated with goat anti-rabbit IgG Alexa Fluor 647 antibody (1:200) and goat anti-mouse IgG Alexa Fluor 594 plus (1:200) in 4% BSA at 4°C overnight. For experiments on Agr2^+^ cells and ionocytes, fixed embryos were dehydrated with 25% methanol/75% PBST, 50% methanol/50% PBST, 75% methanol/25% PBST and 100% methanol for 10 min two times and stored in 100% methanol at -20°C. Embryos were treated with 100% acetone for 15 min at -20°C before being rehydrated with 75% methanol/25% PBST, 50% methanol/50% PBST, 25% methanol/75% PBST and 100% PBT for 5 min four times. Embryos were blocked with 4% BSA in PBT for 1 h at RT. Embryos were incubated with rabbit anti-salmon Agr2 antibody (1:200) and mouse anti-chicken alpha-subunit of Na^+^, K^+^-ATPase antibody (1:200; α5, DSHB Hybridoma) in 4% BSA at 4°C overnight. After washing with PBT for 15 min four times, embryos were incubated with goat anti-rabbit IgG Alexa Fluor 647 antibody (1:200) and goat anti-mouse IgG Alexa Fluor 594 plus (1:200) in 4% BSA at 4°C overnight. After washing with PBT for 15 min four times, embryos were washed with PBS for 5 min three times. Embryos were then stored in antifade reagent (SlowFade Diamond Antifade Mountant with DAPI).

### Double immunofluorescence for BrdU and Agr2

Fixed BrdU-incorporated embryos at 30 and 48 hpf were rehydrated with 75% methanol/25% PBST, 50% methanol/50% PBST, 25% methanol/75% PBST and PBST for 5 min four times. After treatment of 10 μg/mL proteinase K (3 min for 30 hpf and 20 min for 48 hpf) at RT, embryos were washed with PBST for 5–10 min four times, followed by a brief rinse with 2 N HCl and incubation in 2 N HCl for 20 min at RT. After four PBST washes for 5–10 min each, embryos were blocked in 1% blocking reagent for 1 h at RT before incubation with mouse anti-BrdU antibody (1:200, BD Biosciences) and rabbit anti-salmon Agr2 antibody (1:200) in 1% blocking reagents at 4°C overnight. Following six PBST washes for 15 min each, embryos were blocked in 1% blocking reagent for 1 h at RT before incubation with anti-mouse Alexa Fluor 488 (1:200) and anti-rabbit Alexa Fluor 568 (1:200) in 0.5% blocking reagent for 3 h at RT. Embryos were then washed with 1x PBS containing 0.3% Tween 20 for 15 min twice, followed by four PBST washes for 15 min each. Embryos were incubated in Hoechst 33342 in PBST (Invitrogen) for 30 min at RT. After several PBST washes, embryos were stored in 4% PFA in 1x PBS at 4°C before being embedded in 1% low-melting agar for confocal imaging.

### BrdU immunofluorescence and *agr2* fluorescence *in situ* hybridization

BrdU-treated embryos fixed at 26 s and 24 hpf were rehydrated with 75% methanol/25% DEPC-PBST, 50% methanol/50% DEPC-PBST, 25% methanol/75% DEPC-PBST and washed with DEPC-PBST for 5 min four times. After incubation in Hyb^-^ buffer for 5 min and in hybridization buffer for 1 h at 65°C, embryos were hybridized with *agr2* antisense RNA probe (150 ng in 300 μL) in hybridization buffer at 65°C overnight. Embryos were washed at 65°C with pre-heated 25%, 50%, 75% Hyb^-^ buffer in 2x SSC for 10 min each, 2x SSC for 10 min then 1 h, and 0.2x SSC containing 0.1% Tween-20 for 30 min, four times, at 70°C. After two PBST washes for 5 min each, embryos were blocked in 1% blocking reagent in PBST for 1.5 h at RT before incubation with anti-DIG-POD antibody (1:600) in 0.5% blocking reagent in PBST at 4°C overnight. Following six PBST washes for 15 min, embryos were incubated in TSA plus Cy3-tyramide substrate (1:100 diluted in amplification buffer) for 45–60 min at RT. Embryos were then washed with PBST for 10 min twice at RT, followed by incubation in 100 mM glycine for 20 min at RT. After PBST washes for 5 min, five times at RT, embryos were treated with 2 N HCl for 20 min at RT. After PBST washes for 5 min, five times, at RT, embryos were incubated in 1% blocking reagent for 1 h at RT. Embryos were then incubated with anti-BrdU antibody (1:200) in 1% blocking reagent at 4°C overnight. Embryos were washed with PBST for 10 min, six times, followed by incubation with anti-mouse Alexa Fluor 488 (1:200) in 0.5% blocking reagent for 3 h at RT. After six PBST washes for 10 min each, embryos were incubated in Hoechst 33342 in PBST (1:1000) for 30 min at RT, followed by PBST washes. Embryos were stored in 4% PFA in 1 x PBS at 4°C before being embedded in 1% low-melting agar for confocal imaging.

### Transmission electron microscopy and immunogold labeling

*Tg(-6*.*0k agr2*:*EGFP)* embryos at 72 hpf were fixed in prefixative solution (4% PFA and 2.5% glutaraldehyde in 1 x PBS, pH 7.2) at 4°C overnight. Samples were washed with 1x PBS, pH 7.2 for 15 min, three times, at RT with shaking, followed by incubation in postfixative solution (1–2% osmium tetroxide in 1x PBS, pH 7.2) for 2 h at RT in the dark. Samples were washed with 1x PBS for 15 min, two times, at RT with shaking in the dark, followed by dehydration in 30% ethanol/1 x PBS, 50% ethanol/50% 1 x PBS, 70% ethanol/30% 1 x PBS, 80% ethanol/20% 1 x PBS, 90% ethanol/10% 1 x PBS, 95% ethanol/5% 1 x PBS and 100% ethanol for 15 min at RT with shaking in the dark. Samples were dehydrated in 100% ethanol for 15 min, three times, followed by incubation in 100% propylene oxide for 15 min, three times, at RT with shaking in the dark. Samples were incubated in solution containing propylene oxide and Spurr’s resin (3:1) at RT overnight, followed by incubation in a solution containing propylene oxide and Spurr’s resin (1:1) and a solution containing propylene oxide and Spurr’s resin (1:3) for 8 h at RT. Samples were then embedded in 100% Spurr’s resin for 8 h, three times, at RT with shaking, followed by embedding in 100% Spurr’s resin at 60°C for 24 h. Sections (90 nm) were produced using a Leica EM UC7 ultramicrotome and examined using an electron microscope (Tecnai G2 F20S-TWIN, FEI) equipped with a digital camera (UltraScan 1000, Gatan).

For immuno-EM sample preparation [44], embryos were anaesthetized and pre-fixed by 4% PFA for 30 min. Embryos were incubated in 20% BSA at RT and placed into HPF carrier A (200 μm in depth) and carrier B as cap (flat side) to process high-pressure freezing of embryos in EM HPF (HPM100, Leica) to -196°C. Embryos were then transferred to EM AFS2 (Leica) for freeze substitution: Embryos were incubated in 0.2% uranyl acetate in acetone containing 5% water and 4% methanol for 2 to 3 days at -90°C; embryos were warmed to -45°C through a 5°C per day gradient; Embryos were washed with acetone, followed by infiltration with gradient Lowicryl resin (HM20) over one day at -45°C; Embryos were polymerized using UV light for 2 days at -25°C, followed by 2 days at 20°C. For immunogold labeling of GFP, sections (90 nm) were washed with 1 x PBS for 10 min three times, followed by blocking in 2% BSA/1 x PBS for 30 min and incubated with anti-GFP (1:200 or 1:500; Sigma-Aldrich) diluted in 1% BSA/1 x PBS for overnight. After PBS washes for 10 min three times, sections were incubated with Protein A conjugated with 5 nm gold (1: 50; BBI) diluted in PBS for 2 h. After PBS washes for 10 min three times, sections were fixed in 1% glutaraldehyde for 10 min and washed with dH_2_O for 1 min before imaging on an electron microscope (Tecnai G2 F20S-TWIN, FEI) equipped with a digital camera (UltraScan 1000, Gatan).

### Photography, quantification of epidermal mucous cell number and statistical methods

Images of embryos were taken using an AxioCam HRC camera on a Zeiss Axioplan 2 microscope in DIC mode. High-resolution fluorescence images were taken using a Leica TCS-SP5-MP, a Zeiss LSM880 with AiryScan or a Nikon A1R confocal microscope. EMC number was counted from images taken on compound or confocal microscopes using ImageJ software as follows: (i) the image was loaded into ImageJ; (ii) ‘Cell counter’ was selected from ‘Analyze’ items on the Plugins menu; (iii) ‘Initialize’ was selected; and (iv) cell number was determined. All experimental values are presented as mean ± SEM. Two-tailed Student’s *t*-test with unequal variance was conducted in Microsoft Excel to compare experimental groups.

### Real-time quantitative reverse-transcription PCR (RT-qPCR)

DNase I-treated total RNA (1 μg) isolated from AB or *jag1a* mutant embryos at 24 hpf was used to produce first-strand cDNA using oligo (dT) primer and SuperScript III Reverse transcriptase (Invitrogen) at 65°C for 5 min and 50°C for 1 h. qPCRs were conducted by adding cDNAs into 1x SYBR green PCR master mix containing 5 pmole of the respective forward and reverse primers for *18s rRNA*, *jag1a*, *jag1b*, *jag2a* or *jag2b* genes. PCR condition were set to 94°C for 1 min for 1 cycle; and 94°C for 10 s, 60°C for 10 s and 72°C for 10 s for 40 cycles; 72°C for 1 min. Respective primer pair for *18s rRNA* was

F-TCGCTAGTTGGCATCGTTTATG and R-CGGAGGTTCGAAGACGATCA; for *jag1a* was F-CAGGCTGGTCCGGGTTATTT and
R- GGATGAACTGCGACCAGGAA; for *jag1b* was
F- GCTGTGCGGAAACTCTCTCT and R- GCAGCGAAATGCTAACCGAC; for *jag2a* was F- CAGCAGCACAGGACACACTA and
R- GCCTTGGGCCATTCGGATTA; for *jag2b* was

F- TTCACGAGGAGGGCTAGAGT and R- CTGGAGACCCTGAAACCGAG. To calculate the expression level, the comparative cycle threshold (Ct) was used (Roche). △Ct = avg. Ct_GOI_−avg. Ct_18s rRNA_; △△Ct = △Ct_jag mutant_—△Ct_wt_; Fold change = 2 (^-△△Ct^); The biological variance was reported as the SEM, calculated as the standard deviation of multiple samples from two repeats divided by the square root of the number of samples from two repeats.

## Supporting information

S1 Fig *agr2*^*+*^ EMCs and *pvalb8*-positive cells may represent two different populations of epidermal mucous cells. Double fluorescence *in situ* hybridization demonstrates *egfp/agr2* (green) or *pvalb8* (red) expression in different epidermal cells in the trunk and yolk sac of *Tg(-6*.*0 k agr2*:*EGFP)* transgenic embryos at 24 hpf. Arrowhead indicates nonspecific EGFP expression in the intestine. Scale bars, 100 μm.
(TIF)

S2 Fig Proper Notch signaling is required for the differentiation of *agr2*^*+*^ epidermal mucous cells and *pvalb8*-positive cells. **(A).** Substantial reductions in *pvalb8*-positive cell numbers were observed in the trunks and yolks of *mib*^*ta52b*^ and *mib*^*tfi91*^ mutants compared with sibling wild-types at 24 hpf. **(B).** Significant reductions of *agr2*^*+*^ EMC numbers in the trunks and yolks were detected in embryos treated with 100 μM of γ-secretase inhibitor (MK0752) from 10–17 hpf, 10–20 hpf and 10–24 hpf compared to DMSO-treated embryos during 10–24 hpf. **(C).** Substantial decreases in *agr2*^*+*^ EMC numbers in the trunks and yolks were detected in embryos treated with 50 μM of MK0752 from 10–14 hpf or 11–15 hpf but not from 10–13 hpf or 11–14 hpf, as compared to respective DMSO-treated embryos during 10–14 hpf or 11–15 hpf. Arrows, EMCs or *pvalb8*-positive cells. Scale bars, 100 μm. Mean ± SEM. Student’s *t*-test. *p<0.05; ***p<0.001; ns, not significant. Underlying data are available in S2 Data.
(TIF)

S3 Fig Overexpression of *notch1a* but not *notch3* ICD promotes the differentiation of *agr2*^*+*^ epidermal mucous cells and *pvalb8*-positive cells. **(A).** Significant increases in *agr2*^*+*^ EMC and *pvalb8*-positive cell numbers in the trunks and yolks were detected in *notch1a* mRNA-overexpressing embryos at 19 or 24 hpf. **(B).** Concentration-dependent reductions in *agr2*^*+*^ EMC numbers in the trunks and yolks were detected in embryos injected with 10, 50, 100 or 200 pg *notch3* ICD mRNA compared to embryos injected with 200 pg *LacZ* mRNA at 24 hpf. **(C).** Comparable *agr2*^*+*^ EMC numbers were detected in the trunks and yolks of wild types and *notch3* SPMO-injected embryos at 24 hpf. **(D).** Substantial reductions in *agr2*^*+*^ EMC numbers in the trunks and yolks were detected in *notch1a* homozygous mutants injected with 1 or 1.5 ng *notch3* SPMO compared to sibling wild-type embryos injected with the same amount of *notch3* SPMO at 24 hpf. Arrows, EMCs or *pvalb8*-positive cells. Scale bars, 100 μm. Mean ± SEM. Student’s *t*-test. *p<0.05; ***p<0.001; ns, not significant. Underlying data are available in S2 Data.
(TIF)

S4 Fig While *agr2*^*+*^ EMC numbers are not altered in *jag1a* mutants, *jag2b* mutants and *jag2b*-overexpressing embryos, Jagged1a, Jagged1b and Jagged2a are required for the differentiation of *pvalb8*-positive cells. **(A).** Similar *agr2*^*+*^ EMC numbers were observed in the trunks and yolks of *jag1a* homozygous mutants compared to sibling wild-type embryos at 24 hpf. Scale bars, 100 μm. **(B).** Jag1a mutant protein is predicted to contain only MNNL, DSL and six EGF domains with a premature stop codon. However, similar mRNA expression levels for *jag1a*, *jag1b*, *jag2a* and *jag2b* were detected in both *jag1a* homozygous mutants and wild-type embryos by RT-qPCR, using 18s rRNA as a reference gene. **(C).** Similar reduced levels of trunk and yolk *agr2*^*+*^ EMC numbers were detected in wild-types or *jag1a* mutants injected with both *jag1b* and *jag2a* MOs at 24 hpf. **(D).** Similar *agr2*^*+*^ EMC numbers in the trunks and yolks were detected in embryos injected with 500 pg *jag2b* mRNA compared to *LacZ*-injected embryos at 24 hpf. No alterations of *agr2*^*+*^ EMC numbers in the trunks and yolks can be identified among *jag2b* heterozygous, homozygous mutant or sibling wild-type embryos at 24 hpf. **(E).** Significant reductions of *pvalb8*-positive cell numbers were detected in the trunks of *jag2a* mutants or embryos injected with *jag1a* SPMO or *jag1b* UTRMO. Substantial decreases in *pvalb8*-positive cell numbers were observed in the trunks and yolks of embryos injected with *jag1a* UTRMO at 24 hpf. Arrows, EMCs or *pvalb8*-positive cells. Scale bars, 100 μm. Mean ± SEM. Student’s *t*-test. *p<0.05; **p<0.01; ***p<0.001; ns, not significant. Underlying data are available in S2 Data.
(TIF)

S5 Fig Expression pattern of *dlc* and *jag1a*/*jag1b*/*jag2a* and Dlc but not ionocyte or *grhl1*-positive cells participate in the development of *agr2*^+^ epidermal mucous cells. **(A).** Comparable trunk and yolk *agr2*^*+*^ EMC numbers were detected in wild-types and embryos injected with *foxi3a* ATGMO at 24 hpf. Low but significant increases in trunk *agr2*^*+*^ EMC numbers were observed in embryos injected with *foxi3a* ATGMO compared with *foxi3a* misMO-injected control embryos. *atp6v1aa*-positive ionocytes were not detected in embryos injected with *foxi3a* ATGMO unlike those injected with control *foxi3a* misMO and wild-types. **(B).** Comparable trunk and yolk *agr2*^*+*^ EMC numbers were detected in wild-types and embryos injected with *grhl1* ATGMO or *grhl1* SPMO at 24 hpf. Increased *grhl1*-positive cell numbers were observed in the trunks and yolks of embryos injected with *grhl1* ATGMO or *grhl1* SPMO compared with wild-types. **(C).** Co-expression of *dlc* and *jag1a*, *jag1b* or *jag2a* was detected in embryos at 5 s and 10 s. White box indicates the enlarged area. White arrows indicate example cells with colocalization of markers. Yellow arrows indicate example *jag1a*-, *jag1b*- or *jag2a*-expressing cells. **(D).** Similar trunk and yolk *agr2*^*+*^ EMC numbers were detected in sibling wild types and *dlc*^*tm98*^ mutants at 24 hpf. Significant reductions in the numbers of trunk and total *agr2*^*+*^ EMCs were detected in *dlc* morphants compared to wild-type embryos at 24 hpf. Increased *foxi3a*^*+*^ ionocyte numbers were observed in the yolk sac of *dlc* morphants compared to wild types at bud. Increases in the numbers of trunk or yolk and total *agr2*^*+*^ EMCs were detected in 92 or 184 pg *dlc* mRNA-injected embryos compared to *LacZ* mRNA-injected embryos at 24 hpf. Similar trunk and yolk *pvalb8*-positive cell numbers were observed in 92 or 184 pg *dlc* mRNA-injected embryos and *LacZ* mRNA-injected embryos at 24 hpf. Reductions in trunk and yolk *agr2*^*+*^ EMC numbers were detected in embryos injected with *dlc*, *jag1a*, *jag1b* and *jag2a* MOs compared with wild-type embryos at 24 hpf. Arrows, EMCs or *pvalb8*-positive cells. Scale bars, 100 μm. Mean ± SEM. Student’s *t*-test. *p<0.05; **p<0.01; ***p<0.001; ns, not significant. Underlying data are available in S2 Data.
(TIF)

S1 Data Data underlying Figs 2Ac, 2Af, 2Bf, 3Ac, 3Af, 3Bg, 3Bh, 3Bi, 3Bl, 4Ac, 4Af, 4Ai, 4Bc, 4Bd, 4Bg, 4Bh, 4Bk, 4Bl, 4Ce and 6Am. (XLSX)

S2 Data Data underlying S2Ac, S2Af, S2Be, S2Cd, S2Ch, S3Ac, S3Af, S3Bf, S3Cc, S3Dc, S3Df, S4Ac, S4B, S4Cc, S4Cf, S4Dc, S4Dg, S4Ed, S4Ee, S4Ei, S4Ej, S5Ad, S5Bd, S5Dc, S5Df, S5Dk, S5Dl, S5Do and S5Dr Figs. (XLSX)
