# Supplementary figures and images for: Delta/Jagged-mediated Notch signaling induces the differentiation of agr2-positive epidermal mucous cells in zebrafish embryos
Source: PLoS Genet. 2021 Dec 28;17(12):e1009969. doi: 10.1371/journal.pgen.1009969 (PMC8746730; doi:10.1371/journal.pgen.1009969)

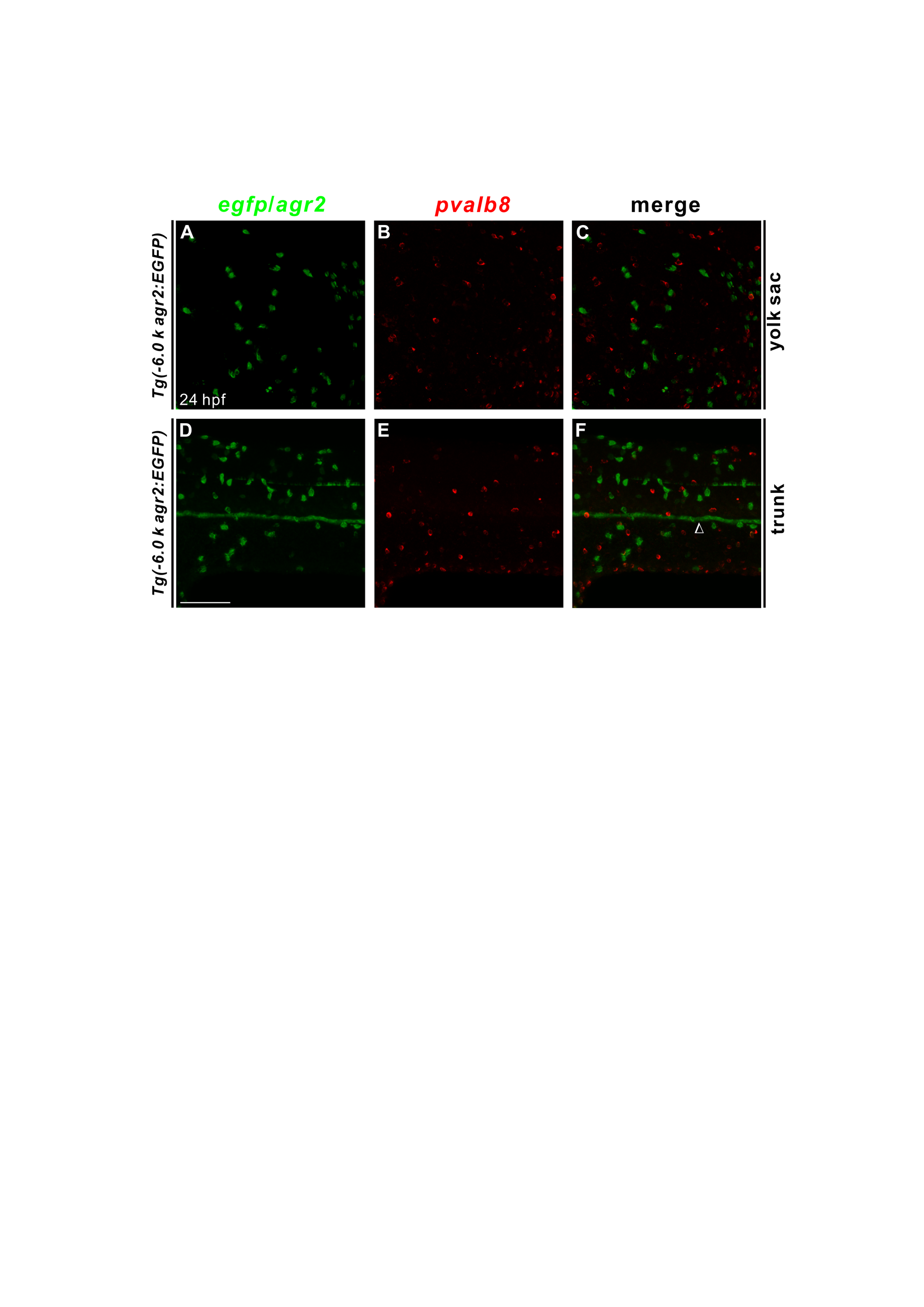

Supplement: S1 Fig — Double fluorescence in situ hybridization demonstrates egfp/agr2 (green) or pvalb8 (red) expression in different epidermal cells in the trunk and yolk sac of Tg(-6.0 k agr2:EGFP) transgenic embryos at 24 hpf. Arrowhead indicates nonspecific EGFP expression in the intestine. Scale bars, 100 μm. (TIF) [file pgen.1009969.s001.tif]

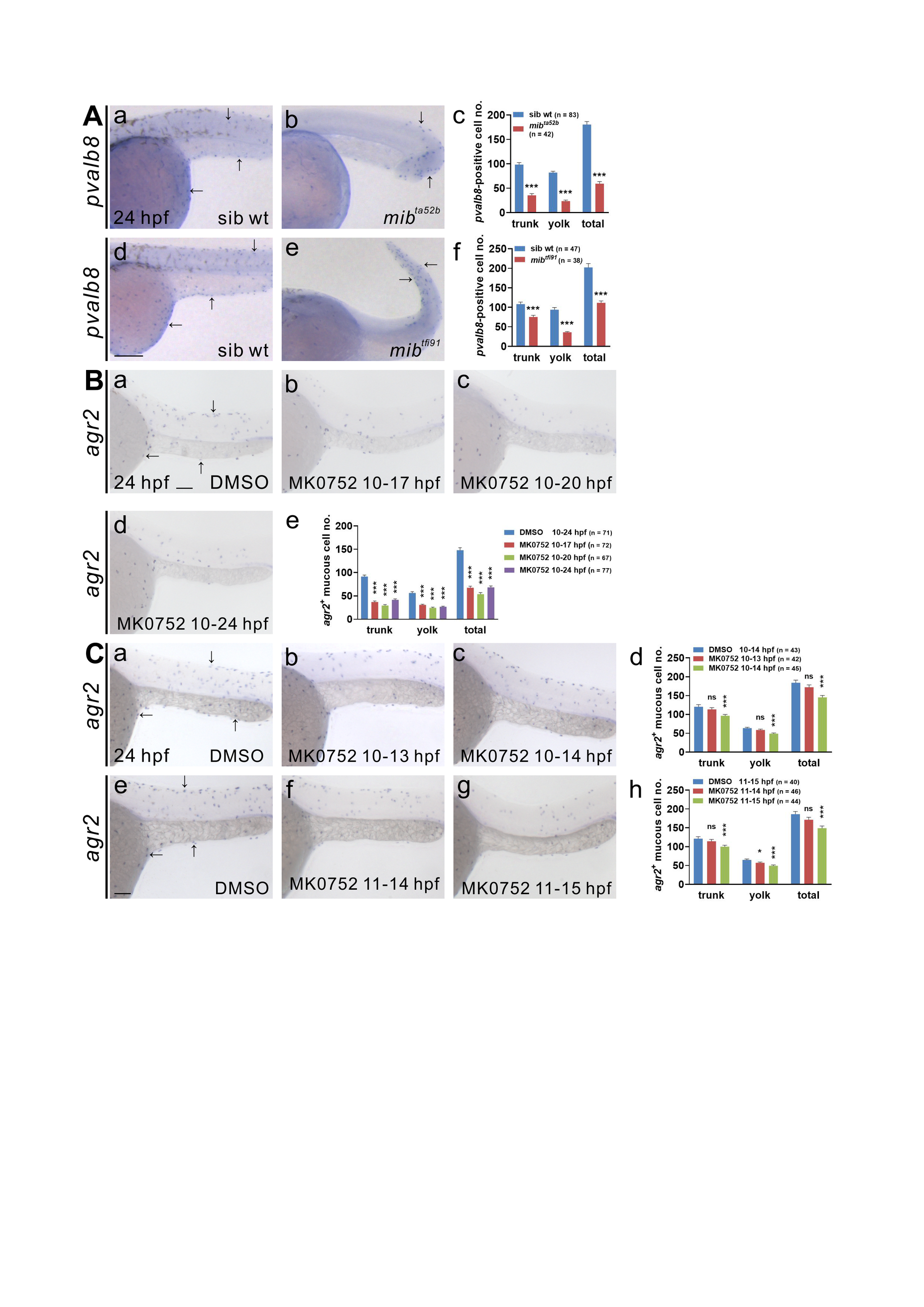

Supplement: S2 Fig — (A). Substantial reductions in pvalb8-positive cell numbers were observed in the trunks and yolks of mibta52b and mibtfi91 mutants compared with sibling wild-types at 24 hpf. (B). Significant reductions of agr2+ EMC numbers in the trunks and yolks were detected in embryos treated with 100 μM of γ-secretase inhibitor (MK0752) from 10–17 hpf, 10–20 hpf and 10–24 hpf compared to DMSO-treated embryos during 10–24 hpf. (C). Substantial decreases in agr2+ EMC numbers in the trunks and yolks were detected in embryos treated with 50 μM of MK0752 from 10–14 hpf or 11–15 hpf but not from 10–13 hpf or 11–14 hpf, as compared to respective DMSO-treated embryos during 10–14 hpf or 11–15 hpf. Arrows, EMCs or pvalb8-positive cells. Scale bars, 100 μm. Mean ± SEM. Student’s t-test. *p<0.05; ***p<0.001; ns, not significant. Underlying data are available in S2 Data. (TIF) [file pgen.1009969.s002.tif]

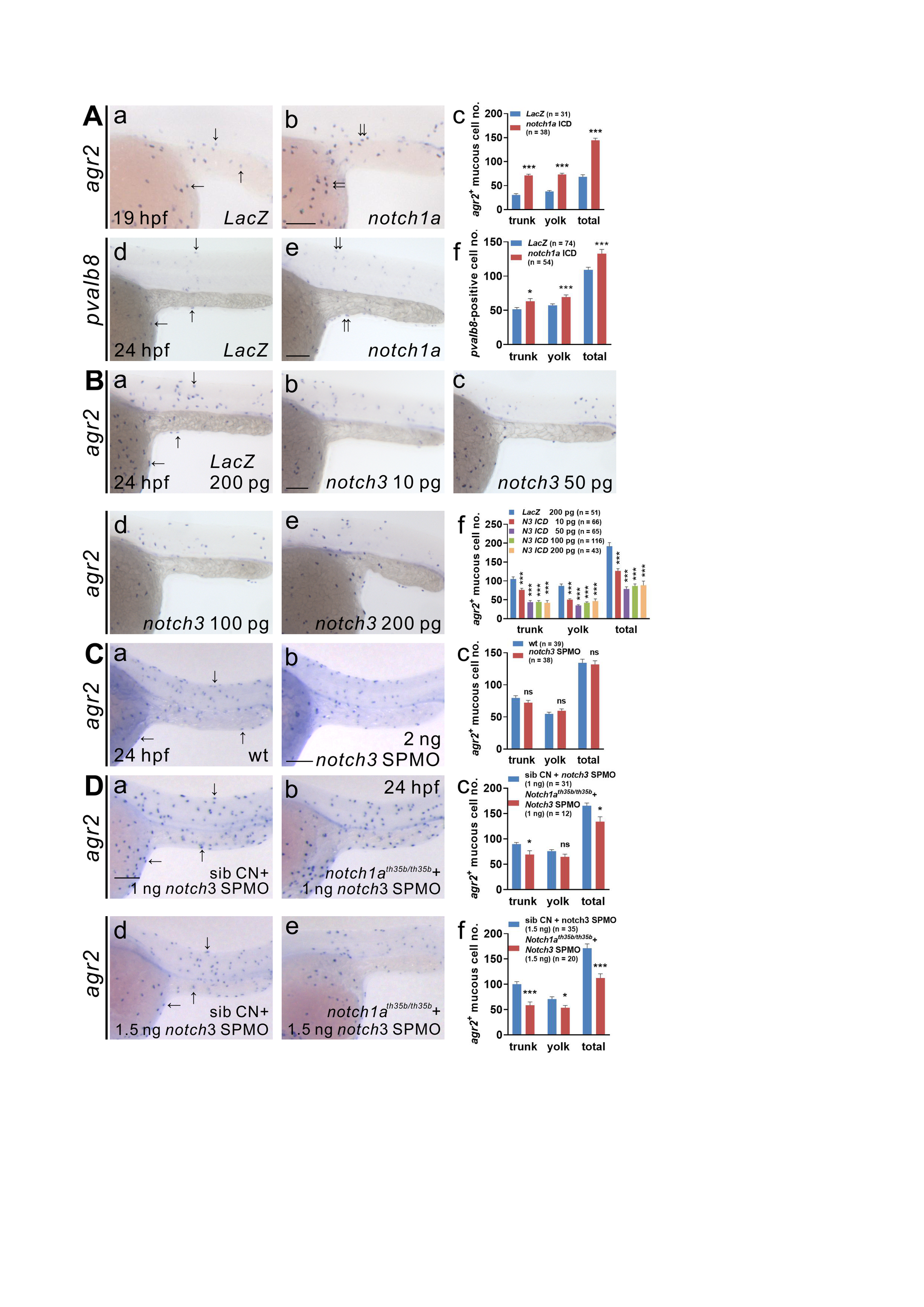

Supplement: S3 Fig — (A). Significant increases in agr2+ EMC and pvalb8-positive cell numbers in the trunks and yolks were detected in notch1a mRNA-overexpressing embryos at 19 or 24 hpf. (B). Concentration-dependent reductions in agr2+ EMC numbers in the trunks and yolks were detected in embryos injected with 10, 50, 100 or 200 pg notch3 ICD mRNA compared to embryos injected with 200 pg LacZ mRNA at 24 hpf. (C). Comparable agr2+ EMC numbers were detected in the trunks and yolks of wild types and notch3 SPMO-injected embryos at 24 hpf. (D). Substantial reductions in agr2+ EMC numbers in the trunks and yolks were detected in notch1a homozygous mutants injected with 1 or 1.5 ng notch3 SPMO compared to sibling wild-type embryos injected with the same amount of notch3 SPMO at 24 hpf. Arrows, EMCs or pvalb8-positive cells. Scale bars, 100 μm. Mean ± SEM. Student’s t-test. *p<0.05; ***p<0.001; ns, not significant. Underlying data are available in S2 Data. (TIF) [file pgen.1009969.s003.tif]

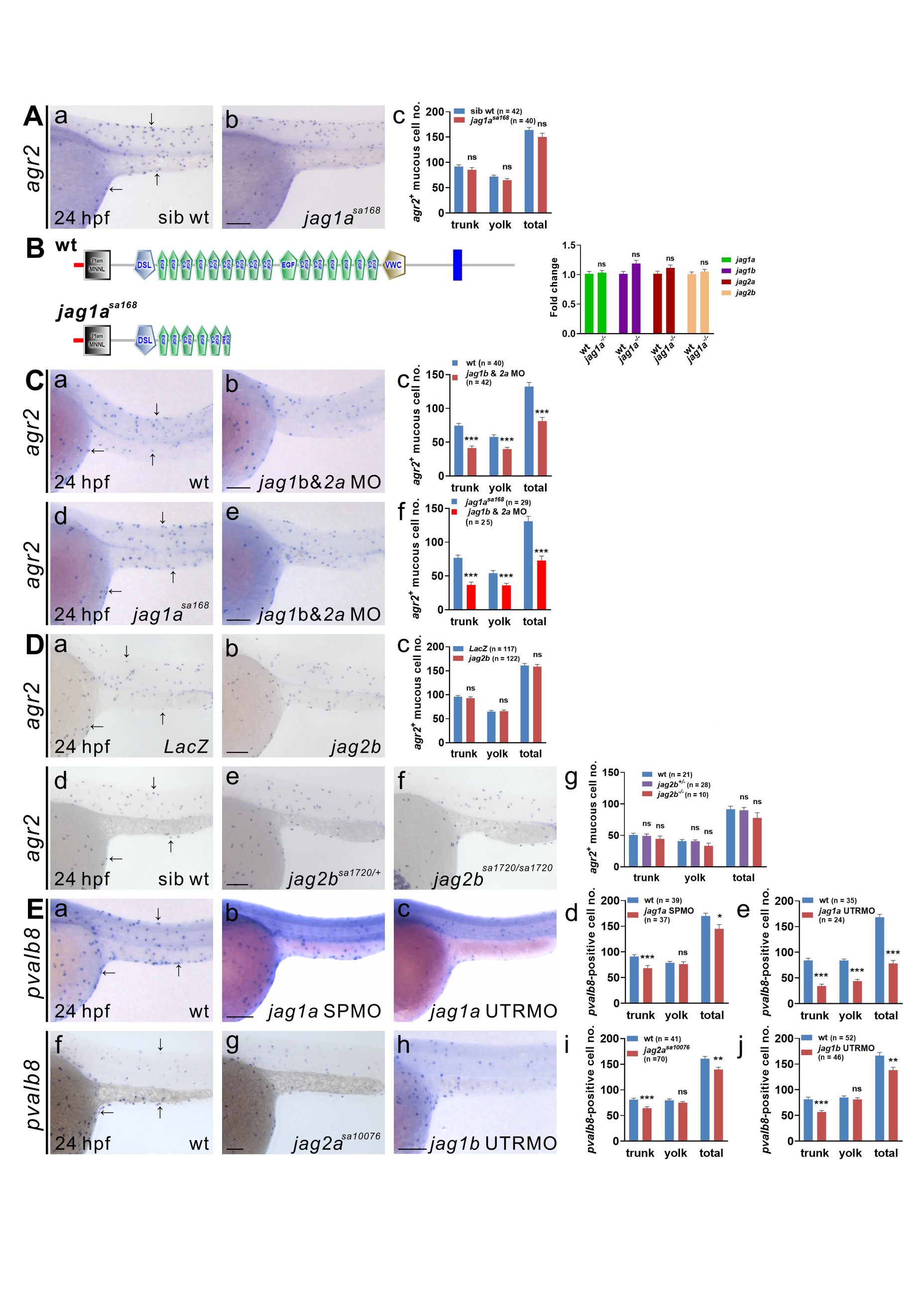

Supplement: S4 Fig — (A). Similar agr2+ EMC numbers were observed in the trunks and yolks of jag1a homozygous mutants compared to sibling wild-type embryos at 24 hpf. Scale bars, 100 μm. (B). Jag1a mutant protein is predicted to contain only MNNL, DSL and six EGF domains with a premature stop codon. However, similar mRNA expression levels for jag1a, jag1b, jag2a and jag2b were detected in both jag1a homozygous mutants and wild-type embryos by RT-qPCR, using 18s rRNA as a reference gene. (C). Similar reduced levels of trunk and yolk agr2+ EMC numbers were detected in wild-types or jag1a mutants injected with both jag1b and jag2a MOs at 24 hpf. (D). Similar agr2+ EMC numbers in the trunks and yolks were detected in embryos injected with 500 pg jag2b mRNA compared to LacZ-injected embryos at 24 hpf. No alterations of agr2+ EMC numbers in the trunks and yolks can be identified among jag2b heterozygous, homozygous mutant or sibling wild-type embryos at 24 hpf. (E). Significant reductions of pvalb8-positive cell numbers were detected in the trunks of jag2a mutants or embryos injected with jag1a SPMO or jag1b UTRMO. Substantial decreases in pvalb8-positive cell numbers were observed in the trunks and yolks of embryos injected with jag1a UTRMO at 24 hpf. Arrows, EMCs or pvalb8-positive cells. Scale bars, 100 μm. Mean ± SEM. Student’s t-test. *p<0.05; **p<0.01; ***p<0.001; ns, not significant. Underlying data are available in S2 Data. (TIF) [file pgen.1009969.s004.tif]

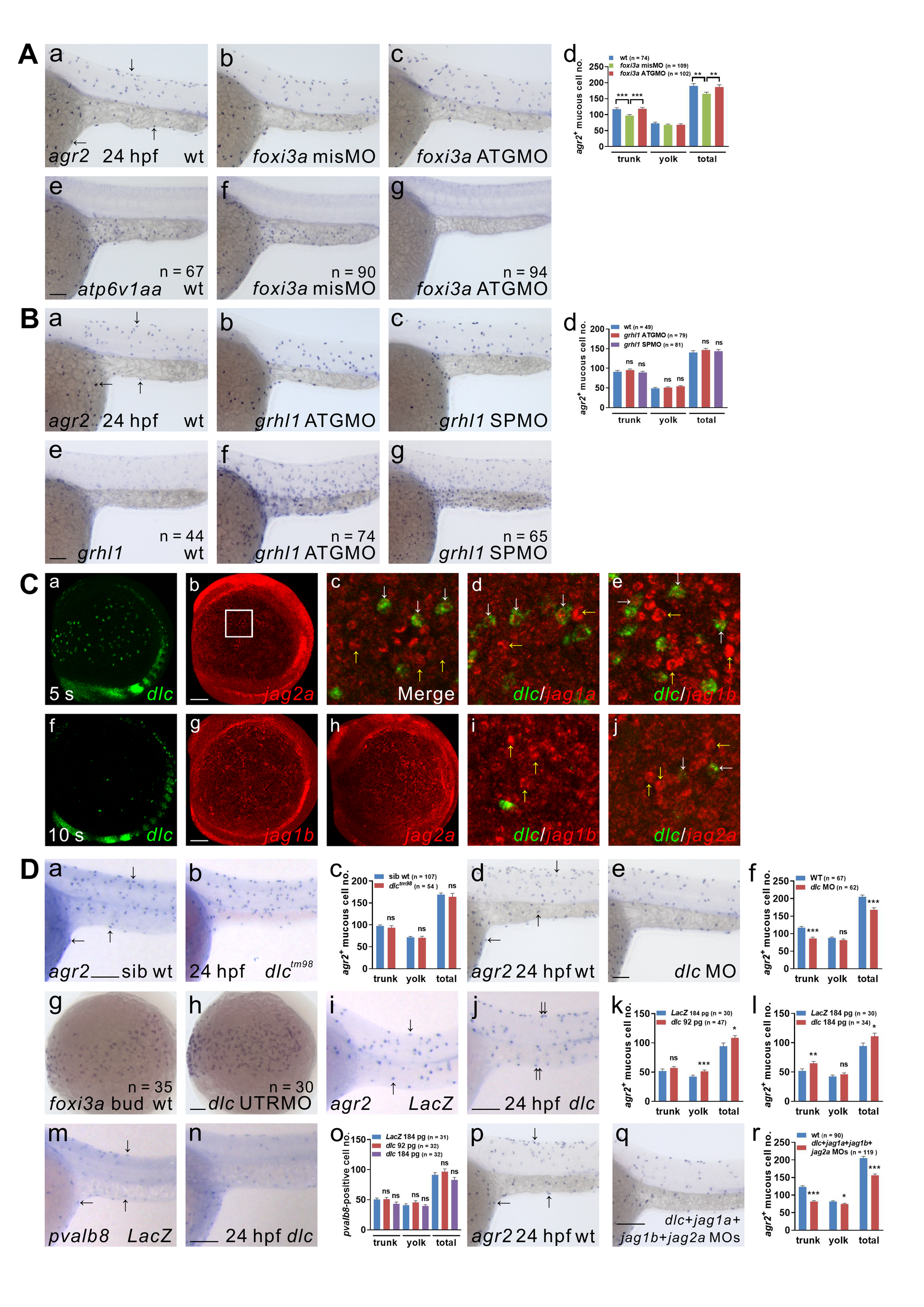

Supplement: S5 Fig — (A). Comparable trunk and yolk agr2+ EMC numbers were detected in wild-types and embryos injected with foxi3a ATGMO at 24 hpf. Low but significant increases in trunk agr2+ EMC numbers were observed in embryos injected with foxi3a ATGMO compared with foxi3a misMO-injected control embryos. atp6v1aa-positive ionocytes were not detected in embryos injected with foxi3a ATGMO unlike those injected with control foxi3a misMO and wild-types. (B). Comparable trunk and yolk agr2+ EMC numbers were detected in wild-types and embryos injected with grhl1 ATGMO or grhl1 SPMO at 24 hpf. Increased grhl1-positive cell numbers were observed in the trunks and yolks of embryos injected with grhl1 ATGMO or grhl1 SPMO compared with wild-types. (C). Co-expression of dlc and jag1a, jag1b or jag2a was detected in embryos at 5 s and 10 s. White box indicates the enlarged area. White arrows indicate example cells with colocalization of markers. Yellow arrows indicate example jag1a-, jag1b- or jag2a-expressing cells. (D). Similar trunk and yolk agr2+ EMC numbers were detected in sibling wild types and dlctm98 mutants at 24 hpf. Significant reductions in the numbers of trunk and total agr2+ EMCs were detected in dlc morphants compared to wild-type embryos at 24 hpf. Increased foxi3a+ ionocyte numbers were observed in the yolk sac of dlc morphants compared to wild types at bud. Increases in the numbers of trunk or yolk and total agr2+ EMCs were detected in 92 or 184 pg dlc mRNA-injected embryos compared to LacZ mRNA-injected embryos at 24 hpf. Similar trunk and yolk pvalb8-positive cell numbers were observed in 92 or 184 pg dlc mRNA-injected embryos and LacZ mRNA-injected embryos at 24 hpf. Reductions in trunk and yolk agr2+ EMC numbers were detected in embryos injected with dlc, jag1a, jag1b and jag2a MOs compared with wild-type embryos at 24 hpf. Arrows, EMCs or pvalb8-positive cells. Scale bars, 100 μm. Mean ± SEM. Student’s t-test. *p<0.05; **p<0.01; ***p<0.001; ns, not significant. Underlying [file pgen.1009969.s005.tif]
